# The EU-AIMS Longitudinal European Autism Project (LEAP): design and methodologies to identify and validate stratification biomarkers for autism spectrum disorders

**DOI:** 10.1186/s13229-017-0146-8

**Published:** 2017-06-23

**Authors:** Eva Loth, Tony Charman, Luke Mason, Julian Tillmann, Emily J. H. Jones, Caroline Wooldridge, Jumana Ahmad, Bonnie Auyeung, Claudia Brogna, Sara Ambrosino, Tobias Banaschewski, Simon Baron-Cohen, Sarah Baumeister, Christian Beckmann, Michael Brammer, Daniel Brandeis, Sven Bölte, Thomas Bourgeron, Carsten Bours, Yvette de Bruijn, Bhismadev Chakrabarti, Daisy Crawley, Ineke Cornelissen, Flavio Dell’ Acqua, Guillaume Dumas, Sarah Durston, Christine Ecker, Jessica Faulkner, Vincent Frouin, Pilar Garces, David Goyard, Hannah Hayward, Lindsay M. Ham, Joerg Hipp, Rosemary J. Holt, Mark H. Johnson, Johan Isaksson, Prantik Kundu, Meng-Chuan Lai, Xavier Liogier D’ardhuy, Michael V. Lombardo, David J. Lythgoe, René Mandl, Andreas Meyer-Lindenberg, Carolin Moessnang, Nico Mueller, Laurence O’Dwyer, Marianne Oldehinkel, Bob Oranje, Gahan Pandina, Antonio M. Persico, Amber N. V. Ruigrok, Barbara Ruggeri, Jessica Sabet, Roberto Sacco, Antonia San José Cáceres, Emily Simonoff, Roberto Toro, Heike Tost, Jack Waldman, Steve C. R. Williams, Marcel P. Zwiers, Will Spooren, Declan G. M. Murphy, Jan K. Buitelaar

**Affiliations:** 1 Sackler Institute for Translational Neurodevelopment, Institute of Psychiatry, Psychology and Neuroscience, King’s College London, De Crespigny Park, Denmark Hill, London, SE5 8AF UK; 2 Department of Forensic and Neurodevelopmental Sciences, Institute of Psychiatry, Psychology and Neuroscience, King’s College London, De Crespigny Park, Denmark Hill, London, SE5 8AF UK; 3 Clinical Child Psychology, Department of Psychology, Institute of Psychiatry, Psychology and Neuroscience, King’s College London, De Crespigny Park, Denmark Hill, London, SE5 8AF UK; 4 Centre for Brain and Cognitive Development, Birkbeck, University of London, Henry Wellcome Building, Malet Street, London, WC1E 7HX UK; 5 Department of Neuroimaging, Institute of Psychiatry, Psychology and Neuroscience, King’s College London, De Crespigny Park, Denmark Hill, London, SE5 8AF UK; 6 Autism Research Centre, Department of Psychiatry, University of Cambridge, Douglas House, 18b Trumpington Road, Cambridge, CB2 8AH UK; 7 Department of Psychology, The School of Philosophy, Psychology, and Language Sciences, University of Edinburgh, Dugald Stewart Building, 3 Charles Street, Edinburgh, EH8 9AD UK; 8 University Campus Bio-Medico, via Álvaro del Portillo, 21, Rome, Italy; 9 Department of Psychiatry, Brain Center Rudolf Magnus, University Medical Center Utrecht, Universiteitsweg 100, 3584 CG Utrecht, The Netherlands; 10 Child and Adolescent Psychiatry, Central Institute of Mental Health, Medical Faculty Mannheim, University of Heidelberg, J5, 68159 Mannheim, Germany; 11 Donders Institute for Brain, Cognition and Behaviour, Radboud University Nijmegen Medical Centre, Kapittelweg 29, 6525 EN Nijmegen, The Netherlands; 12 Department of Child and Adolescent Psychiatry and Psychotherapy, Psychiatric Hospital, University of Zürich, Neumünsterallee 9, 8032 Zürich, Switzerland; 13 Center for Neurodevelopmental Disorders at Karolinska Institutet (KIND), Stockholm, Sweden; 14 Human Genetics and Cognitive Functions Unit, Institut Pasteur, 25 Rue du Docteur Roux, Paris, Cedex 15 France; 15 Centre for Autism, School of Psychology and Clinical Language Sciences, University of Reading, Whiteknights, Reading, RG6 6AL UK; 16 Department of Child and Adolescent Psychiatry, Psychosomatics and Psychotherapy, University Hospital Frankfurt am Main, Goethe University, Deutschordenstrasse 50, 60528 Frankfurt, Germany; 17 Neurospin Centre CEA, Saclay, 91191 Gif sur Yvette, France; 18 Roche Pharma Research and Early Development, Neuroscience, Ophthalmology and Rare Diseases, Roche Innovation Center Basel, Grenzacherstrasse 124, B.001 N.667, CH-4070 Basel, Switzerland; 19 Regulatory Affairs, Product Development, F. Hoffmann-La Roche Pharmaceuticals, Grenzacherstrasse 124, CH-4070 Basel, Switzerland; 20 Department of Neuroscience, Uppsala University, Uppsala, Sweden; 21 Department of Radiology, Icahn School of Medicine at Mount Sinai, NY, USA; 22 Child and Youth Mental Health Collaborative, Centre for Addiction and Mental Health and The Hospital for Sick Children, Department of Psychiatry, University of Toronto, 80, Workman Way, Toronto, ON M6J 1H4 Canada; 23 Center for Applied Neuroscience, Department of Psychology, University of Cyprus, PO Box 20537, 1678 Nicosia, Cyprus; 24 Department of Psychiatry and Psychotherapy, Central Institute of Mental Health, Medical Faculty Mannheim, University of Heidelberg, 68159 Mannheim, Germany; 25 Janssen Research & Development, 1125 Trenton Harbourton Road, Titusville, NJ 08560 USA; 26 Child and Adolescent Neuropsychiatry Unit, Gaetano Martino University Hospital, University of Messina, Via Consolare Valeria 1, I-98125 Messina, Italy; 27 Social, Genetic and Developmental Psychiatry Centre, Institute of Psychiatry, Psychology and Neuroscience, King’s College London, Denmark Hill, London, UK; 28 Department of Child and Adolescent Psychiatry, Institute of Psychology, Psychiatry and Neuroscience, King’s College London, De Crespigny Park, Denmark Hill, London, SE5 8AF UK

**Keywords:** Biomarkers, Cognition, Neuroimaging, MRI, EEG, Eye-tracking, Genetics

## Abstract

**Background:**

The tremendous clinical and aetiological diversity among individuals with autism spectrum disorder (ASD) has been a major obstacle to the development of new treatments, as many may only be effective in particular subgroups. Precision medicine approaches aim to overcome this challenge by combining pathophysiologically based treatments with stratification biomarkers that predict which treatment may be most beneficial for particular individuals. However, so far, we have no single validated stratification biomarker for ASD. This may be due to the fact that most research studies primarily have focused on the identification of mean case-control differences, rather than within-group variability, and included small samples that were underpowered for stratification approaches. The EU-AIMS Longitudinal European Autism Project (LEAP) is to date the largest multi-centre, multi-disciplinary observational study worldwide that aims to identify and validate stratification biomarkers for ASD.

**Methods:**

LEAP includes 437 children and adults with ASD and 300 individuals with typical development or mild intellectual disability. Using an accelerated longitudinal design, each participant is comprehensively characterised in terms of clinical symptoms, comorbidities, functional outcomes, neurocognitive profile, brain structure and function, biochemical markers and genomics. In addition, 51 twin-pairs (of which 36 had one sibling with ASD) are included to identify genetic and environmental factors in phenotypic variability.

**Results:**

Here, we describe the demographic characteristics of the cohort, planned analytic stratification approaches, criteria and steps to validate candidate stratification markers, pre-registration procedures to increase transparency, standardisation and data robustness across all analyses, and share some ‘lessons learnt’. A clinical characterisation of the cohort is given in the companion paper (Charman et al., accepted).

**Conclusion:**

We expect that LEAP will enable us to confirm, reject and refine current hypotheses of neurocognitive/neurobiological abnormalities, identify biologically and clinically meaningful ASD subgroups, and help us map phenotypic heterogeneity to different aetiologies.

**Electronic supplementary material:**

The online version of this article (doi:10.1186/s13229-017-0146-8) contains supplementary material, which is available to authorized users.

## Background

Autism spectrum disorder (ASD) is a life-long neurodevelopmental condition, currently estimated to affect between 1 and 1.5% of children and adults worldwide [1]. Since Kanner’s [2] and Asperger’s seminal case reports [3], diagnostic classification has solely relied on clinical observation, rather than aetiology. Defining symptoms are impairments in social-communication, repetitive and restricted behaviours and interests, and atypical sensory responses (DSM-5 [4]). However, the tremendous clinical, aetiological and genetic heterogeneity among individuals with ASD is now widely recognised. Clinically, individuals with ASD can differ substantially from each other in terms of the quality and severity of core symptoms, level of intellectual ability, co-occurring psychiatric symptoms, and developmental trajectories [5]. Multiple neurocognitive and neurobiological abnormalities have been reported, yet none seem to be shared by all individuals with ASD [6]. Likewise, hundreds of common and rare risk genes have been identified [7]. These diverse genetic as well as environmental risk factors may converge on a smaller number of common molecular pathways, including protein synthesis, synapse development and function, and neuro-immune interaction, which in turn impact brain circuit development and function [8]. However, it is not yet known how different aetiologies and phenotypic diversity at the cellular, molecular, brain systems, cognitive, and/or behavioural level(s) map onto one another.

This heterogeneity has also been a major obstacle to the development of effective treatments. Different people with ASD may have different treatment needs; moreover, most medical treatments may only be *effective* in certain subgroups because similar symptoms may have different biological causes in different individuals. In response to this problem, precision medicine aims to develop treatments based on the understanding of individual differences in the underlying pathophysiology, and then select patients for a particular treatment through use of ‘stratification biomarkers’ [9]. Therefore, a crucial step for this approach is the identification and validation of biomarkers that can parse the condition into distinct (biological) subgroups.

Stratification research in ASD is still in its infancy. In fact, most studies use case-control designs and look for ‘diagnostic biomarkers’. Conceptually, the assumption that ASD involves a variety of pathophysiological mechanisms casts doubt that a truly diagnostic marker—universal and specific to ASD—may exist. Also, the recently developed NIMH Research Domain Criteria (RDoC) Framework suggests that several circuit-based behavioural dimensions may be shared across neurodevelopmental/neuropsychiatric disorders [10]. Methodologically, a mean group difference alone (especially in combination with small effect sizes) does not necessarily indicate that a particular measure would be a good (diagnostic) biomarker for ASD. For example, a test on which the majority of people with ASD falls within 0.5–1 standard deviations of the control group scores would have limited clinical utility in predicting whether someone has ASD or not. Alternatively, a small proportion of individuals with highly atypical scores may drive a mean group difference. On a test with continuous scores, higher scores may be correlated with severity of particular symptoms across ASD and control populations but only indicate risk for ASD above a certain cut-off. This may be more indicative of a potential subgroup, yet within-group variability remains largely unexplored.

In addition, most studies have been hampered by relatively small sample sizes, resulting primarily in lack of power but also the ‘winner’s curse’ (the likelihood of finding exaggerated effects in small studies) [11]. As in many areas of neuroscience [12], in ASD research replication failures are common. Methodological differences, such as different versions of cognitive tests or different neuroimaging analysis approaches, all impact findings and comparability between studies. Therefore, it is often difficult to disentangle whether inconsistencies between findings reflect participant heterogeneity, statistical power, or methods used.

Hence, to identify clinically useful stratification markers, we need to move from group level declarations to a better understanding of *individual* differences, and we need new approaches to identifying potentially (biologically) distinct ASD subgroups. The developmental nature of ASD and the likelihood that there may not be a strict one-to-one correspondence between different levels of analyses add complexity. Large-scale, longitudinal multidisciplinary observational (or ‘natural history’) studies are an important first step to identify stratification markers and track how different biological and clinical profiles are linked over development. This requires collaborative research using a standardised protocol and analysis plan, and stringent statistical approaches [13]. As part of the European Autism Interventions—A Multicentre Study for Developing New Medications (EU-AIMS) consortium (www.eu-aims.eu ) [14, 15], we set up the multicentre Longitudinal European Autism Project (LEAP) to address this challenge.

## Methods

### Overall design of the Longitudinal European Autism Project

LEAP comprises over 800 participants. The case-control study includes approximately 437 individuals with ASD, and 300 controls and uses *an accelerated longitudinal design*. In this design, four cohorts, defined by age and IQ, are recruited concurrently: A. Adults aged 18–30 years; B. Adolescents aged 12–17 years, C. Children aged 6–11 years,—all with IQ in the typical range (75+)—and D. Adolescents and adults aged 12–30 years with ASD and/or mild intellectual disability (ID) (IQ 50–74). Within each schedule, participants are recruited with a male:female ratio 3:1—corresponding to recent estimates of the sex ratio in ASD [16]. The main advantage of the accelerated longitudinal design over a single-cohort longitudinal study lies in the ability to span the age range of interest in a shorter period of time. The cohorts are followed up after 12–24 months using the same core measures (see Table 1). A further twin cohort of *N* = 102 (including 36 monozygotic or dizygotic twin pairs discordant of ASD) is tested at one time point to identify genetic and environmental causes for ASD and to investigate variable expressivity and penetrance of genetic mutations [17].

**Table 1:** LEAP summary of study protocol, by schedule

Level	IA/OA	Domain/task	Time point	Schedule^a^			
				A	B	C	D
Clinical diagnosis							
Level 1	IA	Autism Diagnostic Interview-Revised (ADI-R)^c^	Base	P^b^	P^b^	P^b^	P^b^
Level 1	IA	Autism Diagnostic Observation Schedule (ADOS or ADOS-2)^c^	Base & FU	S^b^	S^b^	S^b^	S^b^
Dimensional measures of ASD symptoms							
Level 2	OA	Social Responsiveness Scale-2nd Edition (SRS-II)^c^	Base & FU	S & P^b^	S & P	P	P
Level 2	OA	Repetitive Behaviour Scale-Revised (RBS-R)^c^	Base & FU	P^b^	P	P	P
Level 2	OA	Short Sensory Profile (SSP)^c^	Base & FU	P^b^	P	P	P
Level 2	OA	Children’s Social Behaviour Questionnaire (CSBQ)^c^ Adults’ Social Behaviour Questionnaire (ASBQ)^c^	Base & FU	– S & P^b^	P –	P –	P –
Level 2	OA	Autism Quotient (AQ), AQ-Adol, AQ-Child^c^	Base & FU	S & P^b^	P	P	P
Level 2	OA	Aberrant Behaviour Checklist	FU	P^b^	P	P	P
Level 2	OA	Adult Routines Inventory (ARI) *or* Childhood Routines Inventory-Revised (CRI-R)	FU	S –	– P	– P	– P
Level 2	OA	Sensory Experiences Questionnaire—short version (SEQ 3.0)	FU	P^b^	P	P	P
Level 2	OA	Global Score of Change	FU	P^b^	P^b^	P^b^	P^b^
Comorbidities							
Level 2	OA	Development and Well-Being Assessment (DAWBA)^c^	Base	S & P^b^	S & P	P	P
Level 2	OA	Strengths and Difficulties Questionnaire (SDQ)^c^	Base & FU	S & P^b^	S & P	P	P
Level 2	OA	DSM-5 ADHD rating scale^c^	Base & FU	S & P^b^	P	P	P
Level 3	OA	Beck Anxiety Inventory (BAI)	Base & FU	S	S	P	P
Level 3	OA	Beck Depression Inventory (BDI)	Base & FU	S	S	P	P
Quality of life/adaptive behaviour							
Level 1	IA	Vineland Adaptive Behaviour Scale-2nd Ed (VABS-2)	Base & FU	P^b^	P^b^	P	P
Level 2	OA	Columbia Impairment Scale (CIS)^c^	Base & FU	S & P^b^	S & P	P	P
	OA	Child Health and Illness Profile (CHIP-CE) *or* World Health Organisation Quality of Life (WHOQOL-BREF)	Base & FU	– S	P –	P –	P –
Medical or psychiatric history							
Level 1	OA	NIH ACE Subject Medical History Questionnaire^c^	Base	S’ or P^b^	P	P	P
Level 2	OA	NIH ACE Family History Form	Base	S’ or P^b^	P	P	P
Level 2	OA	Medical Psychiatric History Perinatal Environmental Risk Questionnaire^c^	FU	P^b^	P	P	P
Level 2	OA	Brief Life Events Questionnaire, anchored in pregnancy	FU	P^b^	P	P	P
Level 2	OA	Children’s Sleep Habits Questionnaire *or* Adult version (FU only)	Base & FU	– S	S & P –	P –	P –
Level 2	IA	Family Medical History Interview	FU	S’ or P^b^	P	P	P
Cognitive and psychological profile							
Level 1	IA	WASI or WISC / WAIS (4 subtests)^c^	Base	S	S	S	S
Level 1	IA	WASI or WISC / WAIS (2 subtests)^c^	FU	S	S	S	S
Level 1	IA	BPVS and RCPM	Base & FU	S	S	S	S
Level 2	OA	HRS-MAT online adaptive IQ test	FU	S	S	S	S
Level 2	IA	Probabilistic reversal learning^c^	Base & FU	S	S	S	S
Level 2	IA	Spatial working memory^c^	Base & FU	S	S	S	S
Level 2	IA	Un/Segmented block design task^c^	Base	S	S	S	S
Level 2	IA	Animated shapes narratives task^c^	Base & FU	S	S	S	S
Level 3	OA	Empathy Quotient	Base & FU	S	P	–	–
Level 3	OA	Systemising Quotient	Base & FU	S	P	–	–
Level 3	OA	Child EQ-SQ	Base & FU	–	–	P	–
Level 3	OA	Toronto Alexithymia Scale (TAS)	Base & FU	S	S	P	P
Level 3	IA	Reading the Mind in the Eyes task (RMET)	Base & FU	S	S	S	S
Level 3	IA	Karolinska Directed Emotional Faces (KDEF)	FU	S	S	S	S
Level 3	IA	Sandbox continuous false belief task	Base & FU	S	S	S	S
Cognitive tests assessed as part of the eye-tracking battery:							
Level 3	IA	Event memory task	FU	S	S	S	S
Level 3	IA	Emotion matching task	Base	S	S	S	S
Level 3	IA	Films expression task	FU	S	S	S	S
Level 3	IA	Visual processing task	FU	S	S	S	S
Level 3	IA	Change detection task	Base or FU	S	S	S	S
Eye-tracking							
Level 2	IA	Natural scenes: static and dynamic^c^	Base & FU	S	S	S	S
Level 2	IA	Gap overlap^c^	Base & FU	S	S	S	S
Level 2	IA	Implicit false belief^c^	Base & FU	S	S	S	S
Level 2	IA	Pupillary light reflex^c^	Base & FU	S	S	S	S
Level 3	IA	Biological motion	Base & FU	S	S	S	S
Neuroimaging							
Level 1	IA	Structural MRI^c^	Base & FU	S	S	S	S
Level 1	IA	FLAIR sequence or localiser sequence MRI^c^	Base & FU	S	S	S	S
Level 2	IA	Diffusion tensor imaging (DTI)^c^	Base & FU	S	S	S	S
Level 2	IA	Resting-state fMRI^c^	Base & FU	S	S	S	S
Level 2	IA	Social/non-social reward fMRI^c^	Base & FU	S	S	S	S
Level 2	IA	Animated shapes theory of mind fMRI^c^	Base & FU	S	S	S	-
Level 2	IA	Flanker Go/No-Go task^c^	Base & FU	S	S	-	-
Level 3	IA	Hariri emotion processing fMRI	Base & FU	S	S	S	-
EEG							
Level 2	IA	Resting state^c^	Base & FU	S	S	S	S
Level 2	IA	Auditory oddball task^c^	Base & FU	S	S	S	S
Level 2	IA	Upright-inverted Faces (gamma)^c^	Base & FU	S	S	S	S
Level 2	IA	Social / non-social videos^c^	Base & FU	S	S	S	S
Biological samples							
Level 2	IA	Blood sample (for genomic analyses)^c^	Base *or* FU	S	S	S	S
Level 2	IA	Saliva (for genomic analyses where blood samples cannot be obtained and for epigenetics)^c^	Base & FU	S	S	S	S
Level 2	IA	Urine (at home, for biochemical biomarkers)^c^	Base *or* FU	S	S	S	S
Level 2	IA	Hair roots (to generate iPSCs)^c^	Base *or* FU	S	S	S	S
Level 2	IA	Head circumference^c^, weight^c^, height^c^	Base & FU	S	S	S	S
Assessment of clinical symptoms and cognition in both biological parents							
Level 3	OA	Social Responsiveness Scale (SRS-2)	Base	P^b^	P^b^	P^b^	P^b^
Level 3	OA	DSM-5 ADHD rating scale	Base	P^b^	P^b^	P^b^	P^b^
Level 3	OA	Beck Anxiety Inventory (BAI)	Base	P^b^	P^b^	P^b^	P^b^
Level 3	OA	Beck Depression Inventory (BDI)	Base	P^b^	P^b^	P^b^	P^b^
Level 3	OA	Adult Routines Inventory (ARI)	FU	P^b^	P	P	P
Level 2	OA	HRS-MAT online adaptive IQ test	FU	P^b^	P	P	P

Level 1 measures are defined as the minimal data set that must be acquired for any one participant to be included in the ‘head count’. Level 2 measures are (a) central to primary and/or secondary study objectives, (b) suitable for the entire targeted participant age and ability range, and (c) have previously shown ASD case-control differences or have been validated in ASD group(s). Level 3 measures are measures that are either (a) more exploratory (e.g. related to novel/emerging hypotheses), (b) less central to the primary or secondary study objectives, and/or (c) only suitable for some schedules. Within each assessment module, in the order of assessments, level 1 and level 2 assessments should be administered before level 3 assessments. Level 3 measures are omitted first in the event of, e.g. participant fatigue or if assessments take considerably longer than average

*ADHD* attention-deficit hyperactivity disorder, *ASD* autism spectrum disorder, *Base* baseline assessment wave, *DSM* Diagnostic and Statistical Manual of Mental Disorders, *fMRI* functional magnetic resonance imaging, *FU* follow-up assessment wave, *IA* investigator administered assessment at the institute, *iPSCs* induced pluripotent stem cells, *NIH ACE* US National Institutes of Health Autism Centers of Excellence, *OA* online assessment, *P* reported by parent, *S* self-reported, *S’* self-reported in the TD adult group in which parents are not enrolled in the study, *sMRI* structural magnetic resonance imaging, *TD* typical development

^a^Schedule A: adults with ASD or TD (aged 18–30 years, with IQ greater than 70); schedule B: adolescents with ASD or TD (aged 12–17 years, with IQ greater than 75); schedule C: children with ASD or TD (aged 6–11 years, with IQ greater than 75); schedule D: adolescents and adults with mild ID (with or without ASD) (aged 12–30 years, with IQ 50–74); schedule E: monozygotic or dizygotic twins (schedule E is not shown but is based on schedules A–C)

^b^ASD groups only

^c^Core measures that were submitted to the European Medicines Agency for QA

**Fig. 1:**
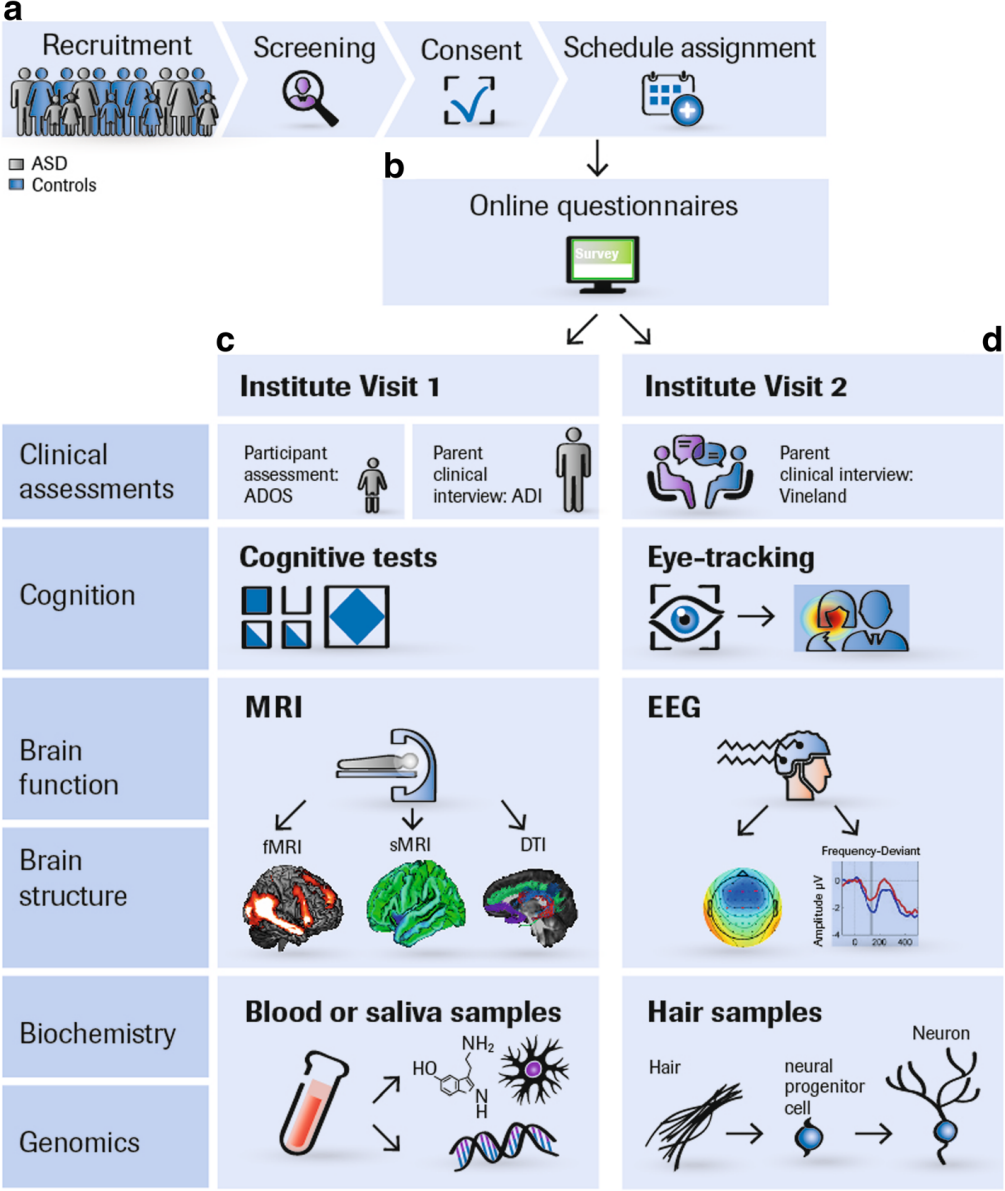
LEAP recruitment and assessment procedures. **a** Participants are concurrently recruited and assessed at seven European study sites: the Institute of Psychiatry, Psychology and Neuroscience, King’s College London, United Kingdom Autism Research Centre at the University of Cambridge, United Kingdom, Radboud University Nijmegen Medical Centre, University Medical Centre Utrecht, the Netherlands, Central Institute of Mental Health, Mannheim, Germany, and the University Campus Bio-Medico, Rome, Italy. Twins are predominantly recruited from the Roots of Autism and ADHD Twin Study in Sweden (RATSS) at Karolinska Institute, Stockholm, Sweden [17]. At each study site, participants with ASD and mild ID are recruited from existing local databases, clinic contacts, and local and national support groups. TD participants are recruited via mainstream schools, flyers (e.g. left at youth centres, colleges, churches, etc.), and existing databases. Participants (or parents) who express interest are sent an information sheet and then screened over the phone for eligibility. If inclusion criteria are confirmed, written consent is obtained and the participant is assigned to a study schedule based on their age and ability level. **b** Parents (as well as adolescents and adults without ID) are sent login details to an online questionnaire (Delosis Ltd., London) to complete at home. **c** and **d** The participant and a parent visit the study centre on two separate occasions within 4 weeks. For participants who travel from far, visits take place on two consecutive days with an overnight stay at a local hotel arranged by the research team. Clinical assessments and interviews are conducted with the participant (e.g. ADOS-2) and a parent (ADI-R, Vineland, Family History Interview). If parents stay with their child during his/her assessments, these interviews are later conducted over the phone. Most cognitive tests are administered using the computerised platform Psytools (Delosis, London Ltd.); some are paper-pencil tests. Eye-tracking is acquired using Tobii-Eye-trackers with a standard acquisition rate of 120 Hz. Tasks are presented interleaved to minimise attentional requirements. Each participant completes a 60–90-min MRI scan session to acquire structural and DTI scans, a resting-state functional MRI scan, and (depending on schedule) one to four task-related fMRI scans. During a training session before the scan, they are instructed to keep still, familiarised with the scanner noise, trained in the functional tasks, and, where possible, are given the opportunity to lie in a mock scanner. During the structural scans, participants watch videos from a video library or DVD brought from home, to make the scan experience more enjoyable. The EEG session tests functional activation during face processing, social and non-social processing, an auditory oddball paradigm (MMN), and resting state. Blood, urine, and saliva samples are taken from the participant and, where possible, both parents for biochemical and genomic analyses. Hair samples are taken to derive induced pluripotent stem cells from selected participants

The project was designed by academic and industry partners and in consultation with the European Medicines Agency (EMA) to increase the chances that stratification biomarkers identified in this study may be qualified to support regulatory decisions for future clinical trials [18]. An overview of our study protocol is given in Table 1 and Additional file 1. Our protocol and standard operation procedures (SOPs) are accessible on https://www.eu-aims.eu/fileadmin/websites/eu-aims/media/EU-AIMS_LEAP/EU-AIMS-LEAP_SOP_StudyProtocol.zip. The study was approved by national and local ethics review boards at each study site and is carried out to Good Clinical Practice (ICH GCP) standards. An overview of the recruitment and study procedures is given in Fig. 1

### Participant selection criteria

In the ASD group, inclusion criteria were an existing clinical diagnosis of ASD according to DSM-IV/ICD-10 or DSM-5 criteria. All psychiatric comorbidities (except for psychosis or bipolar disorder) are allowed as up to 70% of people with ASD have one or more co-occurring psychiatric conditions [19]. Similarly, we include participants on stable medication because 30–50% [20] children and adults with ASD in Europe and up to 70% in the USA are prescribed at least one medication for features, such as aggression, anxiety, hyperactivity, or sleep problems [21]. The intellectual disability (ID) group (defined by IQ between 50 and 74) comprises individuals with both idiopathic and syndromic forms of mild intellectual impairments.

### Clinical characterisation measures in ASD

In the ASD group, diagnosis is confirmed using the combined information of the Autism Diagnostic Interview-Revised (ADI-R [22]) and the Autism Diagnostic Observation Schedule 2 (ADOS-2). Cut-offs on the ADI-R/ADOS-2 are not used as exclusion criteria [23]. To assess dimensional symptom severity across ASD core domains, we used several parent-report instruments with relative emphasis on social-communication [24, 25], repetitive and restricted behaviours [26, 27], sensory processing anomalies [28, 29], and overall autism symptom severity [30, 31] (see Additional file 1). All scales had been validated for the targeted age ranges. In-house translations/back-translations were carried out for some scales. In adolescents and adults with average IQ, companion self-report versions are also included.

We assess a range of psychiatric disorders using the Development and Well-being Assessment (DAWBA, [32]). The most common comorbidities (attention-deficit/hyperactivity disorder (ADHD), depression, anxiety, sleep anomalies) are also assessed at the symptom level using parent and self-report questionnaires. Parental interviews on adaptive behaviour [33] and parent and/ or self-report questionnaires on quality of life [34] provide additional outcome measures.

### Biomarker methodologies

Core measures were selected to be suitable across all age and ability levels to determine whether biomarkers are present only at distinct ages or change with age. We chose measures on which individuals with ASD were previously reported to differ from controls on average and that test several of the most influential neurocognitive (see Table 1) and neurobiological hypotheses of ASD (e.g. differences in brain connectivity [35], excitatory-inhibitory balance [36]). Measures that provide comparable read-outs in animal models and humans were prioritised so that findings can be translated to drug discovery. Some of these measures were taken from the high-risk infant sibling study EUROSIBS (www.eurosibs.eu) allowing us to establish whether some cognitive or neurobiological markers identified in this study also confer risk for developing ASD.

#### Neurocognitive and behavioural markers

Neurocognitive measures included in this study span a wide range of social, motivational, affective, and cognitive domains previously linked to ASD.

*Theory of mind (ToM)* [37] (also called mentalising or mindreading) refers to the ability to represent mental states, such as beliefs, desires, and intentions to predict and explain (others’ and own) behaviour. ToM deficits have been widely regarded as core social-cognitive deficits in ASD and have been hypothesised to underlie, or contribute to, a range of social-communicative impairments. However, severity of impairments has been shown to depend on age and ability level. A useful distinction has been made between explicit (i.e. verbally or cognitively mediated) [38] and implicit or spontaneous ToM [39, 40]. Many high-functioning adolescents and adults with ASD acquire some degree of explicit ToM, whereas abnormalities may primarily consist of persistent deficits in the (typically developmentally earlier emerging) *implicit* theory of mind usage. Therefore, we compare for each participant abnormalities in explicit vs. implicit/spontaneous ToM at the behavioural and (using fMRI) neurofunctional levels because atypical brain processes can persist despite apparently ‘intact’ behaviour.

*Emotion recognition* refers to the ability to infer other people’s emotions from their facial expressions and is therefore critical for many aspects of social-communication. We assess the ability to recognise a range of simple and complex facial expressions in behavioural tests [41] and using eye-tracking [42] and examine neural responses during the processing of facial expressions using fMRI [43].

Another influential theory has proposed that diminished *social motivation* may represent a primary deficit in ASD [44] and is often operationalized as diminished spontaneous attention to social (vs. non-social) information when observing (naturalistic) images or social situations, using eye-tracking. Social motivation deficits may in turn be rooted in diminished social reward sensitivity [45], i.e. the reward value of faces and other social information.

*Executive functions (EF)* is an umbrella term for a set of cognitive processes that rely on prefrontal regions and that include attentional control, inhibitory control, working memory, cognitive flexibility, reasoning, problem solving, and planning. Originally, EF deficits, perhaps notably impairments in cognitive flexibility, were hypothesised to underlie repetitive and restricted behaviours [46, 47]. Whereas evidence for the role of EF in RRBIs is mixed, EF deficits may contribute to both social and non-social ASD symptoms, possibly, by interacting—developmentally or online—with other cognitive systems. Intact EF skills in some individuals may serve as a compensatory mechanism [48] that scaffolds adaptive behaviour [49]. We assess spatial working memory [50] and probabilistic reversal learning [51] using computerised tests and inhibitory control while fMRI blood-oxygen-level dependent (BOLD) responses are recorded.

*Weak central coherence (WCC)* [52] describes a local, detail-focused information processing style, paired with difficulties in global processing, processing information for meaning and in integrating information in context. WCC is thought to pervade different areas of perception and cognition. This account explicitly aims to address islets of talent (in absolute or relative terms) and spared skills. We include the un/segmented block design task as an index of WCC.

*Systemizing* [53] describes a cognitive style characterised by the motivation to predict lawful events (using if-then rules) and observations of input-operation-output relationships and includes good attention to detail. Systemizing is thought to represent a continuum where, on average, males exceed females, and individuals with ASD (both males and females) are shifted to the extreme end of aptitude. It also aims to address relative and absolute strengths in ASD. We assess systemizing using age-appropriage versions of the Systemizing Quotient [54].

*Top-down processing* refers to the fundamental cognitive principle that we use our past experiences and prior knowledge to make sense of the present and predict the future. Top-down processing anomalies have been linked to superior perceptual skills and a more ‘accurate’ or veridical memory in ASD [55].

*Predictive coding* [56, 57] assumes that the brain constantly matches incoming (external) stimuli against a set of (internal) expectations of what will likely happen. Abnormalities in predictive coding may potentially implicated in several facets of repetitive behaviours, sensory processing anomalies, talents as well as social cognitive abilities [58]. Top-down processing is assessed using eye-tracking change detection and event memory tasks; predictability using by studying mismatch negativity derived from an auditory oddball task (using EEG).

Together, this aims to create a comprehensive profile of each participant’s strengths and weaknesses across cognitive domains. Such a cross-domain profile (or composite markers) may potentially be better in predicting symptom severity or functional outcome than the severity of deficits/differences in one single domain. For core domains (e.g. theory of mind), we use convergent methodologies (e.g. behavioural testing, eye-tracking, and fMRI) in the same participant, to identify atypical processes and compensatory mechanisms.

#### Eye-tracking in ASD

Eye-tracking measures can be easily acquired in children and adults with ASD as they are non-invasive and do not require motor responses or language skills. Visual fixation patterns and saccadic control provide a quantitative index of several attentional, perceptual, or social cognitive abnormalities that may be both more specific to particular clinically related features than are questionnaire scores (which typically comprise a composite of several behavioural abnormalities) and more proximal to neurobiological abnormalities. For example, we assess the pupillary light reflex, which largely depends on cholinergic synaptic transmission [59]. We also measure spontaneous visual attention to social and non-social aspects of static and dynamic naturalistic scenes (movie clips). Previous studies found diminished spontaneous attention to the eyes in a high proportion of individuals with ASD from around one year of age through to adults [60, 61]. This behavioural marker of social impairment has been linked to either reduced social reward sensitivity or increased (social) anxiety, which may in turn be mediated by the neuropeptides oxytocin [62] and vasopressin [63], or serotonin [64]. Therefore, changes in visual fixation patterns or saccadic control following a treatment may indicate an initial benefit that presages longer term symptom reduction or behavioural/adaptive changes. It may also provide indications of the neurocognitive mechanisms through which improvements occur.

### Markers of brain structure, function, and connectivity

We use magnetic resonance imaging (MRI) and electroencephalography (EEG) to study differences in brain structure [65], function, and connectivity [35, 66]. These methods are critical to delineating ASD subgroups based on systems-level abnormalities and provide the basis for identifying the mechanisms through which (future) treatments may produce improvements in functioning.

#### MRI and DTI

Across sites, MRI scans are acquired on 3T scanners from different manufacturers (Siemens, Philips, General Electric). We carried out several procedures to optimise structural and functional sequences for the best manufacturer-specific options and to address challenges related to standardisation and quality assurance of multi-site image-acquisition (e.g. use of phantoms, travelling heads).

Measures of total grey and white matter volume provide global descriptors of brain anatomy. Previous neuroimaging studies showed abnormalities in brain development in ASD, with enlarged brain volumes over the first years of life that plateaued across school age and were followed by a more rapid decline from adolescence [65]. This indicates that differences in total brain volume may only reflect risk for ASD at certain developmental stages rather than being causal for the condition. Abnormalities have also been reported in regional brain volumes of children and adults with ASD, including the frontal and temporal cortices, amygdala, hippocampus, caudate nucleus, and cerebellum [67]. These regions support several cognitive, motivational, and emotional functions that are affected in some people with ASD (Table 2). In addition to total and regional brain volumes, we also investigate differences in cortical thickness and cortical surface area, as these anatomical indices have distinct genetic determinants [68], phylogeny, and developmental trajectories [69].

**Table 2:** Neurocognitive domains, underlying brain networks, and neurotransmitter systems

Cognitive domains	Brain network	Neurotransmitter
Theory of mind	Mentalising network [76, 78]; dorsal medial prefrontal cortex, ventro-medial prefrontal cortex, R/L temporal-parietal junction, posterior superior temporal sulcus posterior cingulate cortex/precuneus, R/L anterior temporal lobe, L temporal pole, cerebellar regions	ND
Emotion recognition/emotional reactivity	The corticolimbic circuit; amygdala, medial prefrontal cortex, lateral prefrontal cortex	5-HT, DA, OT, endocannabinoids
Social motivation/social reward sensitivity	Brain reward network: ventral striatum/nucleus accumbens [114], ventral medial prefrontal cortex, orbitofrontal cortex, midbrain; social reward processing further relies on connections to modality specific social information, e.g. right fusiform gyrus implicated in processing faces	OT/AVP, DA
Executive function	Cognitive control network: prefrontal cortex, anterior cingulate cortex, anterior insula, striatum, posterior parietal cortex Working memory: dorsolateral prefrontal (DLPFC), posterior cingulate and parietal cortices	GABA, MAOA
Weak central coherence	Neurobiological underpinnings are not yet fully understood. R DLPFC, parietal cortex, R ventral occipital cortex (vOcc) [115]; broadly consistent with long-range under-connectivity and short-range over-connectivity	ND
Systemizing	Not tested	
Top-down processing/predictive coding	Predictive coding in perception/cognition assumes a hierarchical processing stream and the interplay between feed-forward (bottom-up) and backward (top-down) connections. In a related model predominantly bottom-up-directed gamma-band oscillations are controlled by predominantly top-down-directed alpha-beta-band influences. Not directly tested in ASD.	DA, Ach, NMDA signalling

*Act* acetylcholine, *AVP* arginine vasopressin, *DA* dopamine, *GABA* gamma-aminobutyric acid, *MAOA* monoamine oxidase, *NMDA N*-methyl-d-aspartate, *ND* not determined, *OT* oxytocin, *5-HT* 5-hydroxytryptamine, serotoni

Structural connectivity reflects physical connections between neurons. Its strength depends on the number and efficacy of synapses and in turn affects functional connectivity. We derive indices of structural connectivity both from structural MRI scans and diffusion tensor imaging (DTI). For example, intrinsic grey matter connectivity can be estimated by examining differences in local and global wiring costs [70] and differences in short and long-range white matter tracts using tractography analysis of specific pathways [71].

#### Task-related functional MRI

Four functional MRI paradigms assess neural activation in networks implicated in social and non-social reward processing using an incentive delay task that measures brain reactivity when anticipating a social reward (a woman’s smile) or a monetary reward [72], theory of mind, using an adapted version of the animated shapes task [73], inhibitory control/ conflict monitoring using a Flanker/Go-NoGo task [74], and emotional reactivity to fearful faces [43]. Their known or putative underlying brain networks and implicated neurotransmitter systems are described in Table 2.

The paradigms have been adapted such that each task can be acquired within 5–10 min, as it is challenging for young children and some individuals with ASD (and especially ID) to remain still in the scanner for longer periods of time.

Good test-retest reliability of the fMRI battery was demonstrated in typically developing adults [75, 76]. A pilot study confirmed the feasibility of the tasks for use in children and individuals with ASD. We will use region-of-interest analyses of known areas comprising a particular network (Table 2) as well as exploratory whole-brain analyses in order to investigate potential abnormalities in both activation and functional connectivity within and across tasks.

#### Resting-state fMRI

We use a multi-echo EPI sequence for resting-state fMRI data acquisition. Data are processed using multi-echo independent component analysis and TE-dependent analysis to identify and remove non-neural noise, such as motion artefacts, from the BOLD-signal [77]. This enhances the temporal signal-to-noise ratio in seed connectivity analyses and may increase effect size estimation and statistical power [78]. As motion-related noise can produce spurious correlations throughout the brain [79], this is particularly relevant for connectivity analyses in children and clinical populations who may systematically move more in the scanner than typically developing adults. Multiple functional networks have been identified that are characterised by coherent patterns of intrinsic activity between ‘nodes’ that resemble patterns of activity that are engaged during particular cognitive functions. This includes the ‘default mode network’ and networks implicated in dorsal attention, fronto-parietal control, and motor functions [80]. We aim to identify subgroups with hyper- and hypo-connectivity within and across these networks [81, 82] and examine whether they differ in symptom presentations and/or aetiology.

#### Electroencephalography (EEG)

EEG is a promising biomarker modality with potential clinical utility because of its suitability across broad age and ability ranges, relative low cost, ease of administration, and widespread availability [83, 84]. Its high temporal resolution complements better spatial resolution offered by fMRI. Our EEG methods follow the recent guidelines of recording, analysis, and interpretation of EEG data in autism research [85]. MRI data from the same participants can be used to derive personalised anatomical priors for cortical source reconstruction approaches. We derive two complementary indices: First, using event-related potential (ERP) and event-related oscillation (ERO) paradigms, we study differences in ERP components and EROs that contribute to different sets of neurocognitive processes, such as face processing (P1, N170 components), pre-attentive change detection (mismatch negativity, MMN), or novelty detection (P3a). Second, we use frequency-based analyses to investigate differences in functional activity, variability, and connectivity across all frequency bands (sub-delta to gamma) during resting-state recordings and while passively viewing social and non-social videos. For example, the neurochemical basis of neural firing in the gamma band range depends on interactions between excitatory and inhibitory neurotransmitter concentrations and may therefore serve as a proxy measure of E/I imbalances [86]. Functional connectivity analyses examine differences in short- and long-range synchronisation within and between brain networks and complement connectivity analyses from resting-state fMRI.

#### Biochemical biomarkers

Alterations in the immune system, mitochondrial function, oxidative stress pathways, and several neurotransmitter systems have previously been reported in ASD [87]. For example, increased serotonin blood levels are the most consistently replicated biochemical abnormality found in ASD [88] with approximately 27% of individuals showing significant elevations [89]. As 5-HT elevations appear to be more prevalent in pre- than in post-pubertal ASD samples [90], we will determine the utility of blood serotonin as a biomarker from childhood to adulthood.

#### Genetic markers

We acquire blood samples from the participant, and—where possible—both biological parents, for genomic analyses. First, in collaboration with the Autism Speaks MSSNG project (https://www.mss.ng), we carry out whole-genome sequencing of multiplex (families with two or more individuals with ASD) and simplex families (where only one person has ASD) to assess the combination of inherited and de novo genetic variation (rare and common variants, coding and non-coding variants) that may confer risk for ASD or specific traits linked to ASD. Second, we aim to identify pathways associated with ASD and assign each individual to particular molecular pathways based on their entire mutation profile. In addition, data will be pooled with other international initiatives, to improve the ability to identify new ASD-risk genes.

#### Environmental risk factors

Despite the high heritability of ASD, recent findings indicate that environmental risk factors, notably those acting pre- and perinatally [91, 92], might play a larger role than previously assumed (e.g. maternal immune activation [93], prenatal steroid exposure [94], and gestational diabetes [95]). Therefore, we gather retrospective information on perinatal factors, including any maternal illness/infection, medication, alcohol/drug use, stressful life events, complications during pregnancy/delivery, as well as potentially protective factors, such as the use of vitamins/nutrients.

#### Parent-phenotyping and family psychiatric history

In both biological parents, we administer dimensional measures of the ASD phenotype and assess personality traits linked to ASD (empathising/systematising), commonly co-occurring psychiatric conditions, and IQ.

Using a semi-structured interview, we also obtain comprehensive information on the psychiatric history of first- and second-degree relatives. This addresses the fact that many ASD-risk genes also confer familial vulnerability for a range of other neurodevelopmental/neuropsychiatric disorders and subclinical traits [96].

#### Hair samples

We collect hair roots from participants and first-degree relatives. They are subsequently frozen to generate induced pluripotent stem cells (iPSCs) from selected donors with a particular genomic and/or phenotypic profile. These cell lines help to identify convergent and divergent morphological, cellular, and molecular mechanisms underpinning ASD (subgroups) and for drug screening [97].

#### Central data base/data access

The central database comprises three layers: First, raw data from all recruitment centres (and the on-line questionnaire platform) are uploaded onto the central database using a secured web-interface. Second, for neuroimaging pre-processing and quality control procedures, analysis teams access the raw data via sftp, carry out the necessary analyses locally, and upload quality controlled (QC)/preprocessed data. The final data set is ‘read-only’. A web-based interface enables users to access the database using personalized login details to search, filter, and download data. The EU-AIMS database is currently accessible for internal users but will subsequently be opened to the wider scientific community.

#### Statistical analysis plan

Table 3 outlines the governance structure of EU-AIMS LEAP and describes steps undertaken to increase transparency, standardisation, and reproducibility.

**Table 3:** Governance structure of LEAP, pre-registration, quality control, and reporting of findings

Data analysis is split into expert core analysis groups, broadly defined by data modality (e.g. clinical measures, cognition, EEG, structural MRI, functional MRI, etc.). Each group leads core analyses and coordinates modality-relevant exploratory bottom-up projects. Core analysis groups are closely linked to each other and to ‘cross-cutting’ interest groups (e.g. sex differences, excitatory-inhibitory balance, etc.).
*Registration of projects:* All individual projects (whether they are part of core-analyses or bottom-up projects) are pre-registered on an internal website and shared among the group. Project information includes lead and senior investigators, active collaborators, primary and secondary project goals, and outlines core measures and methodologies. Individual login details to the central EU-AIMS data-base is given upon project review and approval.
*Quality control, standardisation of definitions and analyses:* To maximise coherence and comparability between projects, expert groups lead on modality-specific quality control procedures, which are documented and shared. Where applicable, processing and analysis scripts are also shared to increase transparency and enable replication. Expert groups provide study-wide recommendations, including, for example, a core set of clinical outcome measures, the use of specific covariates, particular analysis approaches pertaining to a given data modality, procedures to correct for multiple-comparisons (e.g. permutations), a priori decisions as to whether/when the data set should be split into a test/replication sample (depending on whether exact or approximate external validation data sets are available). For example, for cognitive analyses, IQ is not recommended to be entered as covariate, as in the present cohort IQ is partially collinear with group status [116]. For all but machine learning approaches, the data set is not split into test/replication (e.g. 70:30%) data sets, as for cross-domain or cross-modal analyses data loss due to missing values is expected, the number and size of empirically derived subgroups are a priori unknown, and therefore the replication data set likely has limited power in replicating findings. In these instances, internal cross-validation strategies (e.g. bootstrapping) should be used. For neuroimaging analyses, core analysis groups carry out centralised pre-processing using a homogeneous automated motion detection algorithm and several quality control procedures, based on consensus agreement on specific parameters, as well as first level values, e.g. of cortical thickness/surface area. For second-level neuroimaging analyses, parametric and non-parametric permutation-based inference methods will be applied depending on the distribution properties of the data. While parametric analyses offer the advantage of efficiency and reproducibility if the underlying distribution assumptions are met, non-parametric approaches offer greater robustness when normality assumptions are violated. These efforts are aimed at increasing consistency between individual projects/analyses, reducing duplication of efforts, and to allow LEAP researchers to benefit from each other’s expertise. In addition, we aim to create a culture that discourages practices such as ‘undisclosed analytic flexibility’, i.e. one uses multiple approaches for one analysis question but only reports the ‘best’ results (‘fishing, *p* value hunting’). However, to strike a balance between standardisation and supporting novel/different approaches, all LEAP researchers can access raw data, use different pre-processing methods or outcome measures, as long as these choices are a priori justified in a project proposal and/or the number of analyses performed are reported and appropriately corrected for.
*Standardised framework for reporting and evaluating biomarkers:* Each project gives summary statistics about effect size, frequency and severity of abnormalities, sensitivity, specificity and—where applicable—cut-offs for dimensional stratification biomarkers. These criteria were identified as a priority for the validation of biomarkers by the European Medicines Agency and follow efforts made to increase consistency in reporting and evaluating case-control studies (see STROBE, http://strobe-statement.org/index.php?id=available-checklists) and clinical trials (see CONsolidation of Standards for Reporting Trials, CONSORT [117]).
*Increased transparency of analyses and findings by depositing a summary of results:* EU-AIMS researchers will deposit for each registered project a summary of results upon completion. The aim is to increase transparency of findings from planned analyses, including ‘negative results’, which are both less frequently written-up for publication and currently more difficult to publish in peer-reviewed journals than positive results [112].

We are using two complementary approaches to identify stratification biomarkers for ASD subtypes. Power calculations are provided in Additional file 2.

### Stratification by participant characterisation criteria

For each measure, we will first test for overall case-control differences and then stratify the sample by age, IQ, sex, and the presence of comorbidities. To investigate age effects in, for example, brain anatomy, resting-state connectivity, or cognitive skills, we first create ‘cross-sectional developmental trajectories’ or ‘growth charts’ for the typically developing (TD) group that test for linear and nonlinear (e.g. quadratic) developmental patterns and determine the typical variability at a particular age [98]. Then, we use confidence intervals around the TD trajectory to assess for each individual with ASD whether, and by how far, he or she falls outside the range of performance expected for their age group. This will help determine whether the abnormality is only detected at a certain developmental stage, or in a subgroup of individuals with ASD across ages. We will also compare several trajectories simultaneously using mixed design linear regression models [98] to establish whether in the ASD group, performance develops with delay or is uneven across domains or component processes. Cross-sectional age-related patterns will be compared to longitudinal (within person) trajectories once follow-up data are available.

Sex differences have previously been reported both in typical development and ASD groups at multiple levels, including serum biomarkers, brain structure and function, several aspects of cognition, and clinical symptom presentation [16]. Likewise, IQ or psychiatric comorbidities may significantly impact on brain and cognitive profile. Hence, we will test diagnosis-by-sex models to identify potentially sex-specific biomarkers and explore whether neurocognitive or neurobiological abnormalities vary with IQ or the presence of psychiatric comorbidities, using both dimensional and categorical approaches. We will also consider potentially mediating (e.g. sleep problems) or moderating factors, such as handedness (lateralisation), medication, and the narrow vs. broader ASD spectrum (ADI-R/ADOS-2 cut-offs).

Progress has also been made in developing *machine learning techniques* for neuroimaging data in order to make clinically relevant predictions [99, 100]. Previous proof-of-concept data show that multivariate pattern classification approaches using structural MRI data discriminated individuals with ASD from healthy controls and non-autistic neurodevelopmental disorders with 90% accuracy [101]. We will apply multivariate approaches to see whether they can discriminate a priori defined subtypes (sex, comorbidities).

To test the potential value of each candidate marker as a ‘surrogate end-point’, we will use correlation and regression analyses to establish whether it relates to or predicts symptom severity (overall, or in a particular domain) or level of adaptive behaviour [102]. For each measure, we will report *p* values adjusted for the number of these core analyses, as well as nominally significant *p* values (to enable comparison with previous studies), effect sizes, and descriptive information on frequency and severity of deficits. For quantitative stratification markers to be of clinical utility, it will be essential to delineate reference values and cut-offs to aid the interpretation of individual scores.

### Stratification by unsupervised, data-driven approaches

The second approach uses data-driven multivariate analysis techniques to identify subgroups based on the pattern of the data itself.

For example, *cluster analyses* are a widely used set of techniques to divide data into prototypical groups based on only the data points and their relationships to one another. Input variables could be multiple cognitive [103], eye-tracking indices [104], EEG values, or a combination of values from different data types. The optimal number of (meaningful) clusters can be determined based on height differences in a cluster tree, while cluster robustness will be evaluated using cross-validation techniques, such as bootstrapping.

*Normative modelling* approaches have also recently been extended to model biological variation across the entire study sample or a typical population [105]. Gaussian process regression is used to predict a set of biological responses (e.g. structural indoor connectivity indices) from a set of clinically relevant covariates (e.g. quantitative cognitive or symptom scores), while estimating predictive confidence for every prediction. This approach enables identification of individuals who are outliers within this distribution and to quantify the degree of deviation in relation to specific symptom domains.

*Functional data analysis (FDA)* takes advantage of trial level data, using curves or trajectories as observational units (for example, eye-tracking gaze paths over time, individual ERP waveforms over the course of the experiment), rather than signals averaged across trials. One recent EEG study reported increased variability of task-related activity in ASD [106]. By combining this approach with a robust multi-level clustering method, recent findings showed distinct learning patterns in particular ASD subgroups [107].

We will also apply recently developed unsupervised techniques to identify meaningful subtypes from the structural and functional neuroimaging data [100]. Further extensions of these methods enable combining different data types (e.g. connectivity indices derived from DTI and resting-state EEG and fMRI) [108], which may further increase specificity/sensitivity of classifiers. Mandatory for these approaches is splitting the data into training and test data sets to avoid ‘overfitting’ and to establish how well the classifiers can predict to which subgroup a new individual belongs.

Molecular biomarkers are potentially of particularly high value to predict treatment response. We will use novel *network-based stratification* approaches similar to those that have recently been validated in cancer research to identify tumour subtypes that are predictive of patient survival or response to therapy [109]. This method integrates genomic information from each individual with functional gene networks (e.g. protein-protein interaction), leveraging prior knowledge to stratify patients in subgroups with specific molecular profiles. We then aim to map those molecular subgroups to biological pathways, structural and functional biomarkers, and clinical symptom profiles.

### Longitudinal follow-up

To test the value of candidate stratification markers in predicting symptom progression, we will initially track the relationship between changes in the neurobiological/ cognitive measure and clinical or behaviour indices at 12–24 months follow-up. In addition, we are seeking additional funding for a third assessment wave to construct for each individual developmental trajectories at multiple levels. This will enable us to ascertain whether subgroups whose (social-communicative, RRBI) symptoms improve, remain the same, or worsen over development [110] differ in terms of their neurobiological/cognitive profile at a given time or the rate of changes (e.g. arrested, uneven across component processes) across particular developmental stages.

### Twin data

Twin data are analysed by applying a statistical framework of multiply adjusted (conditional) linear regressions based on generalised estimations equations (GEE) and allowing both categorical and dimensional ASD outcomes. In addition to the GEE model, an additive genetics, common environment, and unique environmental (ACE) model will be computed to determine heritability estimates. For all analyses, probability estimates for different twin groups will be included, based on the population-based twin cohorts, which allows generalizability of the results.

### Biomarker validation

We will adopt biomarker validation criteria and steps similar to those employed in other biomedical fields, such as oncology, where biomarkers are ‘fit for purpose’, i.e. used in clinical practice [111]. Key criteria against which candidate stratification biomarkers will be validated are performance characteristics (accuracy and reliability) of the measure, reliability in relating to a particular clinical endpoint/clinical symptoms, and its prognostic and/or predictive value. For stratification markers of a priori defined subgroups (e.g. sex, comorbidity), *accuracy* (i.e. sensitivity and specificity, positive and negative predictive value) can be established using receiver operating characteristic (ROC) curves. For subgroups derived from data-driven, unsupervised approaches, external validation is essential as these groups do not necessarily differ in terms of their clinical profile. They may be validated by demonstrating their biological plausibility (i.e. that they have different genetic causes or molecular mechanisms) or functional value (that they differ in terms of their developmental trajectory or respond differentially to a given treatment). The latter cannot be tested in observational studies. Instead, the marker will need to be included in the design of treatment studies or clinical trials in order to compare responders and non-responders in terms of their biomarker characteristics [112]. To ascertain reproducibility, replication in independent samples is essential. For this purpose, we are sharing our protocols and SOPs with other interested international research groups with whom we also have formal data-sharing agreements. They currently include the Australian Cooperative Research Centres (CRC), the French Fondation FondaMental, the Chinese Key 973 program, the Foundation for the National Institutes of Health (FNIH) Autism Biomarker Consortium, and the Province of Ontario Neurodevelopmental Disorders (POND) network. To investigate whether any of these stratification markers are specific to ASD we have aligned several measures with parallel European networks focused on ADHD, obsessive-compulsive disorder, and conduct disorder (MATRICS, TACTICS, NeuroIMAGE [113]).

## Results

### Demographic information of the baseline cohort and assessment rates

Recruitment and assessment of the baseline case-control cohort was carried out between January 2014 (first subject first visit, FSFV) and August 2016, except for schedule D, where recruitment and assessment are ongoing. Follow-up assessments began in September 2015, and all assessments (including schedule D baseline visits) are scheduled to be completed by August 2017. As per protocol, the twins at KI (schedule E) are only seen at one time point. Also, adults at UCBM are only seen at one time point. At the time of writing (08 April 2017), 448 participants have completed their follow-up assessments. Across study sites, retention rates range between 80.3 and 96.2%. (KCL 96.2%, RUNMC 84.7%, UMCU 86.8, UCAM 87.8%, CIMH 80.3%).

#### Cohort characteristics

Tables 4 and 5 give an overview of the baseline sample composition. The ASD and TD groups do not differ in terms of their sex composition overall or by schedule. However, the TD group has on average significantly higher verbal, performance and full-scale IQs than the ASD group (see Charman et al., under review). This was primarily driven by fewer TD individuals with IQs in the lower average range (i.e. 75–90). Age and IQ were not correlated in either group.

**Table 4:** LEAP participant characteristics; case-control cohort, by sex and schedule

		Total		Adults		Adolescents		Children		Mild ID	
		ASD	TD/ID	ASD	TD	ASD	TD	ASD	TD	ASD	ID
Sex	*N*	437	300	142	109	126	94	101	68	68	29
	Males (%)	72.3	65	72.5	67	77	69.1	71.3	61.8	64.7	51.7
	Females (%)	27.7	35	27.5	33	23	30.9	28.7	38.2	35.3	48.3
Age (in years)	M	16.68	17.22	22.79	23.10	14.86	15.33	9.40	9.52	18.09	19.30
	SD	5.80	5.94	3.37	3.27	1.73	1.73	1.58	1.54	4.27	4.97
	Range	6.08–30.60	6.24–30.78	18.02–30.60	18.07–30.78	12.07–17.90	12.04–17.99	6.08–11.97	6.24–11.98	11.50–30.19	12.92–30.24
Full-scale IQ	M	97.61	104.57	103.99	109.15	101.59	106.58	105.29	111.46	65.84	63.39
	SD	19.74	18.26	14.82	12.60	15.68	13.18	14.76	12.69	7.70	8.00
	Range	40^a^–148	50–142	76–148	76–142	75–143	77–140	74–148	76–142	40^a^–74	50–74

*ASD* autism spectrum disorder, *TD* typically developing, *Mild ID* intellectual disability

^a^There are 3 individuals with a full-scale IQ <50

**Table 5:** LEAP participant characteristics; twin cohort

		MZ/ DZ twin pairs (at least one ASD sibling)		TD twins	
		Twin 1: ASD	Twin 2: ASD or TD	Twin 1	Twin 2
Diagnosis Sex	N	36	36	15	15
	ASD (%)	100	66.7	71.5	65.8
	Males:female (%)	61.5:38.4	70.2:29.9	56.3:43.7	53.3:46.7
Age (in years)	M	15.9	15.9	16.8	16.9
	SD	4.5	4.5	2.9	2.9
	Range	6–27	6–27	12–21	12–21
Full-scale IQ	M	94.1	94.2	103.6	103.7
	SD	19.5	19.0	13.7	12.6
	Range	40–122	58–130	76–124	79–126

#### Data acquisition rates

For MRI measures, acquisition rates in the ASD group ranged from 93% (structural scan) to 47% (emotion processing functional scan) and in the TD group from 96 to 60%. This difference in acquisition rates between sequences is largely explained by our hierarchy of level 1 (core) to level 3 (optional) measures, such that level 3 measures were always omitted first (e.g. when scanning started late, technical problems were encountered, or the participant expressed that he/she wanted to stop the scan session). For cognitive tests, acquisition rates ranged between 92–86% in the ASD group and 97–87% in the TD group. For EEG measures, acquisition rates in the ASD group ranged between 83–75% and 80–79% in the TD group. These lower acquisition rates for EEG measures reflect the fact that one site (UCAM) did not acquire EEG data. For eye-tracking, acquisition rates of the four main task sets ranged between 91–86% and 91–87% in the ASD and TD groups, respectively. Two tasks that were later added to the protocol (change detection, emotion matching) had lower acquisition rates. Blood samples were acquired in 68% of ASD participants and 73% of TD participants and ‘trios’ (i.e. participant, biological father, and biological mother) in 29% of people with ASD. Saliva was acquired in those individuals who did not wish to give a blood sample, 39% of people with ASD and 30% of TD people. Urine was given by 82% of participants in both the ASD and TD groups, and hair samples in 43% of people with ASD and 35% of people with TD.

Additional file 3 shows the data acquisition rates across the different assessment modalities and split by group and schedules.

## Discussion

### ‘Lessons learnt’ and future directions

The scale and level of integrated phenotypic and genomic characterisation of the LEAP cohort is unprecedented in ASD research. Within a 5-year period, we have been able to comprehensively assess a large cohort of children, adolescents, and young adults from 6 to 30 years at two time points. As the final sample slightly exceeds the original recruitment target, the study (accounting for missing data and data loss after QC procedures) has the power to detect subgroups with medium effect sizes.

However, we also wish to acknowledge limitations of the study, and ‘share lessons’ learnt regarding the study design, recruitment, and data acquisition.

#### Study design

It is important to allow sufficient time for study set-up, piloting of tasks, development of standard operating procedures (SOP), and training prior to commencing data collection. This includes dedicated periods for developing QC and data processing pipeline SOPs. As most grants have a timeline of 5 years, the development of new measures, such as cognitive tests, neuroimaging paradigms or clinical scales (including piloting, validation, establishing test-retest reliability) is difficult to integrate in this schedule. For (European) studies carried out in different countries in which different languages are spoken, time for translation/back-translation (of some measures) needs to be factored in. Therefore, in LEAP, we predominantly used well-established tests (but adapted tasks where needed, carried out test-retest reliability studies of the fMRI tasks, and optimised acquisition protocols)—perhaps at the cost of including more novel tests or emergent domains. LEAP is one of the few biomarker studies that covers a broad age range. Therefore, one selection criterion of tasks was their suitability across the entire age/ability range of the cohort. An alternative approach could be to use tasks with different age-appropriate versions; however, with regard to the most prominent ASD neurocognitive domains, such tests are currently largely missing.

We deliberately used few participant exclusion criteria, and LEAP is one of the few biomarker—and more specifically neuroimaging—studies that includes people with ASD and mild intellectual disability. However, we did exclude individuals with moderate, severe, and profound ID. This decision was largely made because at the start of the study, it was deemed difficult to use some of the same measures/tests in individuals with severe ID, including MRI scanning (without sedation). However, we are currently carrying out a companion single-site study (EU-AIMS Synaptic Gene Project, SynaG) that focuses on individuals with rare monogenic forms of ASD (e.g. SHANK3), most of whom have severe-to-profound ID.

#### Recruitment

We recruited individuals with an existing clinical diagnosis of ASD using a range of different avenues. This, together with the fact that people with moderate-to-profound ID were excluded and that we deliberately over-recruited females means that the cohort may not be representative of the ASD population. Recruitment of individuals with mild ID has proven to be challenging. We also found that a relatively high proportion of individuals with ASD that were recruited into schedule D based on an initial screener had IQ higher than 70 but lower adaptive behaviour. Likewise, recruitment of a population-representative TD control group, specifically, individuals with below-average IQ (e.g. between 75 and 90) was found to be a challenge. Future studies may require specific recruitment strategies to recruit this population.

#### Data acquisition

In order to collect high-quality data, building in standardisation at every level on the way is crucial. In our experience, investing in joint training for research teams across sites and face-to-face meetings has proven invaluable. We established QC procedures for data entry and checking and included a proportion of double data entry to identify systematic errors/inconsistencies across sites. Furthermore, communication is key, including Principal Investigators, Postdocs, PhD students, and Research Assistants. For example, our study operations manager hosts weekly telephone calls with researchers from all study sites to ensure consistency in data acquisition, identify problems early, and trouble shoot. Periodically checking/analysing data (not only after the pilot phase) is essential to ensure that no errors creep in after, for example, a script update or scanner upgrade, and to detect any errors as early as possible. We found facebook and twitter to be useful for recruitment calls and to tell families about study progress, events, relevant papers or findings, and to stay in contact. However, we believe that our very good to excellent retention rates are primarily due to the ability of the research teams to make the study visits comfortable and a positive experience for the participants and families, despite our comprehensive study protocol.

## Conclusion

We expect that planned core analyses will enable us to confirm, reject, and refine existing neurocognitive and neurobiological hypotheses (e.g. regarding case-control differences). Data-driven, unsupervised techniques hold promise to identifying ‘new’ ASD subgroups based on their shared cognitive and/or neurobiological/neurochemical or genetic profile. By integrating diverse multi-level analyses, we hope to attain a better picture of how different aetiologies (e.g. genetically driven molecular subgroups) and neurobiological and clinical heterogeneity map onto one another. Validation and qualification of these markers will be crucial to determine their potential usefulness as ‘enrichment markers’ for clinical trials. Ultimately, the clinical utility of a stratification marker will lie in its value to predict, for a particular person, how their symptoms progress, and whether or not they may benefit from a specific treatment or intervention. We expect that the findings generated in this study will be important steps in bringing us closer to developing precision medicines approaches for ASD.

## Additional files

Additional file 1: Summary of study protocol, by schedule*. (DOCX 72 kb)

Additional file 2: Data acquisiton rates of baseline assessments, by data modality. (DOCX 786 kb)

Additional file 3: Power calculations. (DOCX 1019 kb)

## Abbreviations

ADHD: Attention-deficit/hyperactivity disorder
ADI-R: Autism Diagnostic Interview-Revised
ADOS: Autism Diagnostic Observation Schedule
AQ: Autism Spectrum Quotient
ASBQ: Adult Social Behaviour Questionnaire
ASD: Autism Spectrum Disorder
BOLD: Blood-oxygen-level dependent
CIMH: Central Institute of Mental Health
DAWBA: Development and Well-being Assessment
DTI: Diffusion tensor imaging
E/I: Excitatory-Inhibitory
EEG: Electroencephalography
EF: Executive Function
EMA: European Medicines Agency
EPI: Echo-planar imaging
ERO: Event-related oscillation
ERP: Event-related potential
EU-AIMS: European Autism Interventions—A Multicentre Study for Developing New Medications
FDA: Functional data analysis
fMRI: Functional magnetic resonance imaging
ICD-10: International Statistical Classification of Diseases and Related Health Problems, 10th Revision
ID: Intellectual disability
iPSC: Induced pluripotent stem cells
IQ: Intelligent quotient
KCL: King’s College London
LEAP: Longitudinal European Autism Project
MMN: Mismatch negativity
MRI: Magnetic resonance imaging
MSSNG: https://www.mss.ng
P: Parent-report
RBS-R: Repetitive Behavior Scale-Revised
RDoC: Research Domain Criteria
ROC: Receiver Operating Characteristic
RRB: Repetitive and Restricted Behaviours
RUNMC: Radboud University Nijmegen Medical Centre
S: Self-report
Sftp: Secure file transfer protocol
SOP: Standard operating procedure
SRS-2: Social Responsiveness Scale, 2nd Edition
TD: Typically Developing
ToM: Theory of mind
UCAM: Autism Research Centre, University of Cambridge
UCBM: University Campus Bio-Medico
UMCU: University Medical Centre Utrecht
WCC: Weak central coherence
