# Supplementary material for: The EU-AIMS Longitudinal European Autism Project (LEAP): design and methodologies to identify and validate stratification biomarkers for autism spectrum disorders
Source: Mol Autism. 2017 Jun 23;8:24. doi: 10.1186/s13229-017-0146-8 (PMC5481887; doi:10.1186/s13229-017-0146-8)
Supplement: Supplementary file 1 — Summary of study protocol, by schedule*. (DOCX 72 kb) [file 13229_2017_146_MOESM1_ESM.docx]

**Additional file 1: Summary of Study protocol, by Schedule***

| **Level** | **IA / OA** | **Domain / Task** | **Time-point** | **Schedule*** | | | | | |
| --- | --- | --- | --- | --- | --- | --- | --- | --- | --- |
|  |  |  |  | **A** | **B** | | | **C** | **D** |
|  |  | Clinical diagnosis | | | | | | | |
| Level 1 | IA | - Autism Diagnostic Interview-Revised (ADI-R) [1]^§^ | Base | P^%^ | P^%^ | | | P^%^ | P^%^ |
| Level 1 | IA | - Autism Diagnostic Observation Schedule (ADOS or ADOS-2)[2] ^§^ | Base & FU | S^%^ | S^%^ | | | S^%^ | S^%^ |
|  |  | Dimensional measures of ASD symptoms | | | | | | | |
| Level 2 | OA | - Social Responsiveness Scale-2^nd^ Edition (SRS-II) [3]^§^ | Base & FU | S & P^%^ | S & P | | | P | P |
| Level 2 | OA | - Repetitive Behaviour Scale-Revised (RBS-R) [4]^§^ | Base & FU | P^%^ | P | | | P | P |
| Level 2 | OA | - Short Sensory Profile (SSP) [5]^§^ | Base & FU | P^%^ | P | | | P | P |
| Level 2 | OA | - Children’s Social Behaviour Questionnaire (CSBQ) [6]^§^ - Adults’ Social Behaviour Questionnaire (ASBQ) ^§^ | Base & FU | -  S & P^%^ | P  - | | | P  - | P  - |
| Level 2 | OA | - Autism Quotient (AQ) [7], AQ-Adolescent [8], AQ-Child[9]^§^ | Base & FU | S & P^%^ | P | | | P | P |
| Level 2 | OA | - Aberrant Behaviour Checklist [10] | FU | P^%^ | P | | | P | P |
| Level 2 | OA | - Adult Routine Inventory (ARI) [11] *or*   Child Routine Inventory (CRI-R) [11] | FU | S  - | -  P | | | -  P | -  P |
| Level 2 | OA | - Sensory Experiences Questionnaire – short version (SEQ 3.0) [12] | FU | P^%^ | P | | | P | P |
| Level 2 | OA | - Global Score of Change | FU | P^%^ | P^%^ | | | P^%^ | P^%^ |
|  |  | Comorbidities | | | | | | | |
| Level 2 | OA | - Development and Well-Being Assessment (DAWBA) [13]^§^ | Base | S & P^%^ | S & P | | | P | P |
| Level 2 | OA | - Strengths and Difficulties Questionnaire [14] (SDQ)^§^ | Base & FU | S & P^%^ | S & P | | | P | P |
| Level 2 | OA | - DSM-5 ADHD rating scale^§^ | Base & FU | S & P^%^ | P | | | P | P |
| Level 3 | OA | - Beck Anxiety Inventory [15] | Base & FU | S | S | | | P | P |
| Level 3 | OA | - Beck Depression Inventory [16] | Base & FU | S | S | | | P | P |
|  |  | Quality of life / Adaptive Behaviour |  |  | |  |  | |  |
| Level 1 | IA | - Vineland Adaptive Behaviour Scale [17] | Base & FU | P^%^ | P^%^ | | | P | P |
| Level 2 | OA | - Columbia Impairment Scale (CIS) [18]^§^ | Base & FU | S & P^%^ | S & P | | | P | P |
|  | OA | - Child Health and Illness Profile (CHIP-CE) [19] *or*   World Health Organisation Quality of Life (WHOQOL-BREF) [20] | Base & FU | -  S | P  - | | | P  - | P  - |
|  |  | Medical or Psychiatric History |  |  | |  |  | |  |
| Level 1 | OA | - NIH ACE Subject Medical History Questionnaire^§^ | Base | S’ or P^%^ | P | | | P | P |
| Level 2 | OA | - NIH ACE Family History Form | Base | S’ or P^%^ | P | | | P | P |
| Level 2 | OA | - Medical Psychiatric History Perinatal Environmental Risk Questionnaire, in house^§,$^ | FU | P^%^ | P | | | P | P |
| Level 2 | OA | - Brief Life Events Questionnaire, anchored in pregnancy [21]^$^ | FU | P^%^ | P | | | P | P |
| Level 2 | IA | - EU-AIMS Family Medical History Interview, in-house | FU | S’ or P^%^ | P | | | P | P |
| Level 2 | OA | - Children’s Sleep Habits Questionnaire [22] *or*   Adult version (FU only) | Base & FU | -  S | S & P  - | | | P  - | P  - |
|  |  | Cognitive and Psychological Profile |  |  | |  |  | |  |
| Level 3 | OA | - Empathy Quotient [23] | Base & FU | S | P | | | - | - |
| Level 3 | OA | - Systemising Quotient [24] | Base & FU | S | P | | | - | - |
| Level 3 | OA | - Child EQ-SQ [25] | Base & FU | - | - | | | P | - |
| Level 3 | OA | - Toronto Alexithymia Scale [26] | Base & FU | S | S | | | P | P |
| Level 1 | IA | - WASI [27] or WISC [28] / WAIS [29] (4 subtests)^§^ | Base | S | S | | | S | S |
| Level 1 | IA | - WASI or WISC / WAIS (2 subtests)^§^ | FU | S | S | | | S | S |
| Level 1 | IA | - BPVS [30] and RCPM [31] | Base & FU | S | S | | | S | S |
| Level 2 | OA | - HRS-MAT online adaptive IQ test [32] | FU | S | S | | | S | S |
| Level 2 | IA | - Probabilistic reversal learning [33]^§^ | Base & FU | S | S | | | S | S |
| Level 2 | IA | - Spatial working memory [34] ^§^ | Base & FU | S | S | | | S | S |
| Level 2 | IA | - Visual search^§^ | FU | S | S | | | S | S |
| Level 2 | IA | - Un/Segmented block design task [35]^§^ | Base & FU? | S | S | | | S | S |
| Level 3 | IA | - Reading the Mind in the Eyes task [36] [37] | Base & FU | S | S | | | S | S |
| Level 3 | IA | - Karolinska Directed Emotional Faces (KDEF) [38] | FU | S | S | | | S | S |
| Level 3 | IA | - Sandbox continuous false belief task [39] | Base & FU | S | S | | | S | S |
| Level 2 | IA | - Animated shapes narratives task [40]^§^ | Base & FU | S | S | | | S | S |
|  |  | Neuroimaging |  |  | |  |  | |  |
| Level 1 | IA | - Structural MRI^§^ | Base & FU | S | S | | | S | S |
| Level 1 | IA | - FLAIR sequence or localiser sequence MRI^§^ | Base & FU | S | S | | | S | S |
| Level 2 | IA | - Diffusion tensor imaging (DTI)^§^ | Base & FU | S | S | | | S | S |
| Level 2 | IA | - Resting-state fMRI^§^ | Base & FU | S | S | | | S | S |
| Level 2 | IA | - Social/non-social reward fMRI, based on [41]^§^ | Base & FU | S | S | | | S | S |
| Level 2 | IA | - Animated shapes theory of mind fMRI, based on [42]^§^ | Base & FU | S | S | | | S | - |
| Level 2 | IA | - Flanker Go/No-Go task, based on [43]^§^ | Base & FU | S | S | | | - | - |
| Level 3 | IA | - Hariri emotion processing fMRI [44] | Base & FU | S | S | | | S | - |
|  |  | EEG |  |  | |  |  | |  |
| Level 2 | IA | - Resting state^§^ | Base & FU | S | S | | | S | S |
| Level 2 | IA | - Auditory oddball task, in house^§^ | Base & FU | S | S | | | S | S |
| Level 2 | IA | - Upright-inverted Faces (gamma), [45]^§^ | Base & FU | S | S | | | S | S |
| Level 2 | IA | - Social / non-social videos [46]^§^ | Base & FU | S | S | | | S | S |
|  |  | Eye-tracking |  |  | |  |  | |  |
| Level 2 | IA | - Natural scenes: static and dynamic [47], [48], ^§^ | Base & FU | S | S | | | S | S |
| Level 2 | IA | - Gap overlap [49]^§^ | Base & FU | S | S | | | S | S |
| Level 2 | IA | - Implicit false belief [50]^§^ | Base & FU | S | S | | | S | S |
| Level 2 | IA | - Pupillary light reflex^§^ | Base & FU | S | S | | | S | S |
| Level 3 | IA | - Biological motion [51] | Base & FU | S | S | | | S | S |
| Level 3 | IA | - Event memory task [52] | FU | S | S | | | S | S |
| Level 3 | IA | - Emotion matching, based on [53] | Base | S | S | | | S | S |
| Level 3 | IA | - Films expression task, in house | FU | S | S | | | S | S |
| Level 3 | IA | - Visual processing task, in house | FU | S | S | | | S | S |
| Level 3 | IA | - Change detection task [54] | Base & FU | S | S | | | S | S |
|  |  | Biological samples |  |  | |  |  | |  |
| Level 2 | IA | - Blood sample (for genomic analyses)^§^ | Base or FU | S | S | | | S | S |
| Level 2 | IA | - Saliva (for genomic analyses where blood samples cannot be obtained and for epigenetics)^§^ | Base & FU | S | S | | | S | S |
| Level 2 | IA | - Urine (at home, for biochemical biomarkers)^§^ | Base or FU | S | S | | | S | S |
| Level 2 | IA | - Hair roots (to generate iPSCs)^§^ | Base or FU | S | S | | | S | S |
| Level 2 | IA | - Head circumference^§^, weight^§^, height^§^ | Base & FU | S | S | | | S | S |
|  |  | Assessment of clinical symptoms and cognition in both biological parents | | | | | | |  |
| Level 3 | OA | - Social Responsiveness Scale (SRS-2) | Base | P^%^ | P^%^ | | | P^%^ | P^%^ |
| Level 3 | OA | - DSM-5 ADHD rating scale | Base | P^%^ | P^%^ | | | P^%^ | P^%^ |
| Level 3 | OA | - Beck Anxiety Inventory (BAI)[15] | Base | P^%^ | P^%^ | | | P^%^ | P^%^ |
| Level 3 | OA | - Beck Depression Inventory (BDI) [16] | Base | P^%^ | P^%^ | | | P^%^ | P^%^ |
| Level 3 | OA | - (Short) Adult Routines Inventory (ARI) [11] | FU | P^%^ | P | | | P | P |
| Level 2 | OA | - HRS-MAT online adaptive IQ test [32] | FU | P^%^ | P | | | P | P |

ADHD, attention-deficit hyperactivity disorder; ASD, autism spectrum disorder; Base, baseline assessment wave; DSM, Diagnostic and Statistical Manual of Mental Disorders; fMRI, functional magnetic resonance imaging; FU, follow-up assessment wave; IA, investigator administered assessment at the institute; iPSCs, induced pluripotent stem cells; NIH ACE, US National Institutes of Health Autism Centers of Excellence; OA, online assessment; P, reported by parent; S, self-reported; S’, self-reported in the TD adult group in which parents are not enrolled in the study; sMRI, structural magnetic resonance imaging; TD, typical development.

*Schedule A: adults with ASD or TD (aged 18–30 years, with IQ greater than 70); Schedule B: adolescents with ASD or TD (aged 12–17 years, with IQ greater than 70); Schedule C: children with ASD or TD (aged 6–11 years, with IQ greater than 70); Schedule D: adolescents and adults with mild ID (with or without ASD) (aged 12–30 years, with IQ 50–70); Schedule E: monozygotic or dizygotic twins (Schedule E is not shown but is based on Schedules A–C).

%ASD groups only; §Core measures that were submitted to the European Medicines Agency for Qualification Advice.

^$^Biological mother only

**References**

1. Rutter M, Le Couteur A, Lord C: **Autism Diagnostic Interview - Revised.** *Western Psychological Services* 2003.

2. Lord C, Rutter M, DiLavore PC, Gotham K, Bishop SL: **Autism Diagnostic Observation Schedule, Second Edition (ADOS-2).** *Western Psychological Services* 2012.

3. Constantino JN, Gruber CP: **Social Responsiveness Scale (SRS).** *Western Psychological Services* 2005.

4. Bodfish JW, Symons FJ, Parker DE, Lewis MH: **Varieties of repetitive behavior in autism: comparisons to mental retardation.** *J Autism Dev Disord* 2000, **30:**237-243.

5. Tomchek SD, Dunn W: **Sensory processing in children with and without autism: a comparative study using the short sensory profile.** *Am J Occup Ther* 2007, **61:**190-200.

6. Hartman CA, Luteijn E, Serra M, Minderaa R: **Refinement of the Children's Social Behavior Questionnaire (CSBQ): an instrument that describes the diverse problems seen in milder forms of PDD.** *J Autism Dev Disord* 2006, **36:**325-342.

7. Baron-Cohen S, Wheelwright S, Skinner R, Martin J, Clubley E: **The autism-spectrum quotient (AQ): evidence from Asperger syndrome/high-functioning autism, males and females, scientists and mathematicians.** *J Autism Dev Disord* 2001, **31:**5-17.

8. Baron-Cohen S, Hoekstra RA, Knickmeyer R, Wheelwright S: **The Autism-Spectrum Quotient (AQ)--adolescent version.** *J Autism Dev Disord* 2006, **36:**343-350.

9. Auyeung B, Baron-Cohen S, Wheelwright S, Allison C: **The Autism Spectrum Quotient: Children's Version (AQ-Child).** *J Autism Dev Disord* 2008, **38:**1230-1240.

10. Aman MG, Singh NN, Stewart AW, Field CJ: **The aberrant behavior checklist: a behavior rating scale for the assessment of treatment effects.** *Am J Ment Defic* 1985, **89:**485-491.

11. Evans DW, Uljarevic M, Lusk LG, Loth E, Frazier T: **Development of two new dimensional measures of restricted and repetitive behaviors in parents and children.** *Journal of the American Academy of Child and Adolescent Psychiatry* in press, **doi: 10.1016/j.jaac.2016.10.014**.

12. Ausderau K, Sideris J, Furlong M, Little LM, Bulluck J, Baranek GT: **National survey of sensory features in children with ASD: factor structure of the sensory experience questionnaire (3.0).** *J Autism Dev Disord* 2014, **44:**915-925.

13. Goodman R, Ford T, Richards H, Gatward R, Meltzer H: **The Development and Well-Being Assessment: description and initial validation of an integrated assessment of child and adolescent psychopathology.** *J Child Psychol Psychiatry* 2000, **41:**645-655.

14. Goodman R: **The Strengths and Difficulties Questionnaire: A Research Note.** *Journal of Child Psychology and Psychiatry* 1997, **38:**581-586.

15. Beck AT, Steer RA: **Beck Anxiety Inventory Manual.** *San Antonio: Harcourt Brace and Co* 1993.

16. Beck A, Steer R, Brown: **Manual for the Beck Depression Inventory-II.** *San Antonio, TX: Psychological Corporation* 1996.

17. Sparrow SS, Ciccetti DV, D.A. B: **Vineland Adpative Behavior Scales, Second Edition (Vineland-II).** *American Guidance Services Publishing* 2005.

18. Bird HR, Shaffer D, Fisher P, M.S. G: **The Columbia Impairment Scale (CIS): Pilot findings on a measure of global impairment for children and adolescents.** *International Journal of Methods in Pyschiatric Research* 1993, **3:**167-176.

19. Riley AW, Forrest C, Starfield B, Rebok G, Robertson J: *Child Health and Illness Profile - Child Edition (CHIP-CE).* Baltimore, MD: The John Hopkins University; 2001.

20. World Health Organization Group TW: **Development of the World Health Organization WHOQOL-BREF quality of life assessment. .** *Psychol Med* 1998, **28:**551-558.

21. Brugha TS, Cragg D: **The List of Threatening Experiences: the reliability and validity of a brief life events questionnaire.** *Acta Psychiatr Scand* 1990, **82:**77-81.

22. Owens J, Maxim R, McGuinn M, Nobile C, Msall M, Alario A: **Television-viewing habits and sleep disturbance in school children.** *Pediatrics* 1999, **104:**e27.

23. Baron-Cohen S, Wheelwright S: **The empathy quotient: an investigation of adults with Asperger syndrome or high functioning autism, and normal sex differences.** *J Autism Dev Disord* 2004, **34:**163-175.

24. Baron-Cohen S, Richler J, Bisarya D, Gurunathan N, Wheelwright S: **The systemizing quotient: an investigation of adults with Asperger syndrome or high-functioning autism, and normal sex differences.** *Philos Trans R Soc Lond B Biol Sci* 2003, **358:**361-374.

25. Auyeung B, Wheelwright S, Allison C, Atkinson M, Samarawickrema N, Baron-Cohen S: **The children's Empathy Quotient and Systemizing Quotient: sex differences in typical development and in autism spectrum conditions.** *J Autism Dev Disord* 2009, **39:**1509-1521.

26. Taylor GJ, Ryan D, Bagby RM: **Toward the development of a new self-report alexithymia scale.** *Psychother Psychosom* 1985, **44:**191-199.

27. Wechsler D: *Wechsler Abbreviated Scale of Intelligence - Second Edition (WASI-II).* London: Pearson; 2011.

28. Wechsler D: *The Wechsler Intelligence Scale for Children - Fourth Edition (WISC-IV).* London: Pearson; 2004.

29. Wechsler D: *Wechsler Adult Intelligence Scale - Fourth Edition (WAIS-IV).* London: Pearsons; 2008.

30. Dunn LM, Dunn DM, Styles B, National Foundation for Educational Research, Sewell J: **British Picture Vocabulary Scale: 3rd Edition (BPVS3).** *Granada* 2013.

31. Evans RB, Marmorston J: **Scoring Raven's Coloured Progressive Matrices to Differentiate Brain Damage.** *J Clin Psychol* 1964, **20:**360-364.

32. Hansen Research Services: *Matrix Adaptive test (HRS-MAT).* 2014.

33. den Ouden HE, Daw ND, Fernandez G, Elshout JA, Rijpkema M, Hoogman M, Franke B, Cools R: **Dissociable effects of dopamine and serotonin on reversal learning.** *Neuron* 2013, **80:**1090-1100.

34. Sjowall D, Roth L, Lindqvist S, Thorell LB: **Multiple deficits in ADHD: executive dysfunction, delay aversion, reaction time variability, and emotional deficits.** *J Child Psychol Psychiatry* 2013, **54:**619-627.

35. Shah A, Frith U: **Why do autistic indiviudals show superior performance on the Block Design task?** *Journal of Child Psychology and Psychiatry* 1993, **34**.

36. Baron-Cohen S, Wheelwright S, Hill J, Raste Y, Plumb I: **The 'Reading the Mind in the Eyes' test revised version: A study with normal adults, and adults with Asperger Syndrome or high-functioning autism.** *Journal of Child Psychology and Psychiatry* 2001**:**in print.

37. Holt RJ, Chura LR, Lai MC, Suckling J, von dem Hagen E, Calder AJ, Bullmore ET, Baron-Cohen S, Spencer MD: **'Reading the Mind in the Eyes': an fMRI study of adolescents with autism and their siblings.** *Psychol Med* 2014, **44:**3215-3227.

38. Sucksmith E, Allison C, Baron-Cohen S, Chakrabarti B, Hoekstra RA: **Empathy and emotion recognition in people with autism, first-degree relatives, and controls.** *Neuropsychologia* 2013, **51:**98-105.

39. Begeer S, Bernstein DM, van Wijhe J, Scheeren AM, Koot HM: **A continuous false belief task reveals egocentric biases in children and adolescents with autism spectrum disorders.** *Autism* 2012, **16:**357-366.

40. Abell F, Happe F, Frith U: **Do triangles play tricks? Attribution of mental states of animated shapes in normal and abnormal development.** *Cognitive Development* 2000, **15:**1-16.

41. Delmonte S, Balsters JH, McGrath J, Fitzgerald J, Brennan S, Fagan AJ, Gallagher L: **Social and monetary reward processing in autism spectrum disorders.** *Mol Autism* 2012, **3:**7.

42. Castelli F, Frith C, Happe F, Frith U: **Autism, Asperger syndrome and brain mechanisms for the attribution of mental states to animated shapes.** *Brain* 2002, **125:**1839-1849.

43. Blasi G, Goldberg TE, Elvevag B, Rasetti R, Bertolino A, Cohen J, Alce G, Zoltick B, Weinberger DR, Mattay VS: **Differentiating allocation of resources and conflict detection within attentional control processing.** *Eur J Neurosci* 2007, **25:**594-602.

44. Hariri AR, Mattay VS, Tessitore A, Kolachana B, Fera F, Goldman D, Egan MF, Weinberger DR: **Serotonin transporter genetic variation and the response of the human amygdala.** *Science* 2002, **297:**400-403.

45. Tye C, Mercure E, Ashwood KL, Azadi B, Asherson P, Johnson MH, Bolton P, McLoughlin G: **Neurophysiological responses to faces and gaze direction differentiate children with ASD, ADHD and ASD+ADHD.** *Dev Cogn Neurosci* 2013, **5:**71-85.

46. Jones EJ, Venema K, Lowy R, Earl RK, Webb SJ: **Developmental changes in infant brain activity during naturalistic social experiences.** *Dev Psychobiol* 2015, **57:**842-853.

47. Vo ML, Smith TJ, Mital PK, Henderson JM: **Do the eyes really have it? Dynamic allocation of attention when viewing moving faces.** *J Vis* 2012, **12**.

48. Elsabbagh M, Gliga T, Pickles A, Hudry K, Charman T, Johnson MH, Team B: **The development of face orienting mechanisms in infants at-risk for autism.** *Behav Brain Res* 2013, **251:**147-154.

49. Elsabbagh M, Fernandes J, Jane Webb S, Dawson G, Charman T, Johnson MH: **Disengagement of Visual Attention in Infancy Is Associated with Emerging Autism in Toddlerhood.** *Biol Psychiatry* 2013.

50. Senju A, Southgate V, White S, Frith U: **Mindblind eyes: an absence of spontaneous theory of mind in Asperger syndrome.** *Science* 2009, **325:**883-885.

51. Annaz D, Campbell R, Coleman M, Milne E, Swettenham J: **Young children with autism spectrum disorder do not preferentially attend to biological motion.** *J Autism Dev Disord* 2012, **42:**401-408.

52. Loth E, Gómez JC, Happé F: **Do high-functioning people with autism spectrum disorder spontaneously use event knowledge to selectively attend to and remember context-relevant aspects in scenes?** *Journal of Autism and Developmental Disorders* 2011, **41:**945-961.

53. Palermo R, O'Connor KB, Davis JM, Irons J, McKone E: **New tests to measure individual differences in matching and labelling facial expressions of emotion, and their association with ability to recognise vocal emotions and facial identity.** *PLoS One* 2013, **8:**e68126.

54. Loth E, Carlos Gomez J, Happe F: **Detecting changes in naturalistic scenes: contextual inconsistency does not influence spontaneous attention in high-functioning people with autism spectrum disorder.** *Autism Res* 2008, **1:**179-188.
