# Supplementary material for: The EU-AIMS Longitudinal European Autism Project (LEAP): design and methodologies to identify and validate stratification biomarkers for autism spectrum disorders
Source: Mol Autism. 2017 Jun 23;8:24. doi: 10.1186/s13229-017-0146-8 (PMC5481887; doi:10.1186/s13229-017-0146-8)

**Additional file 2. Data acquisiton rates of baseline assessments, by data modality.**

1. **MRI**

(a) by group


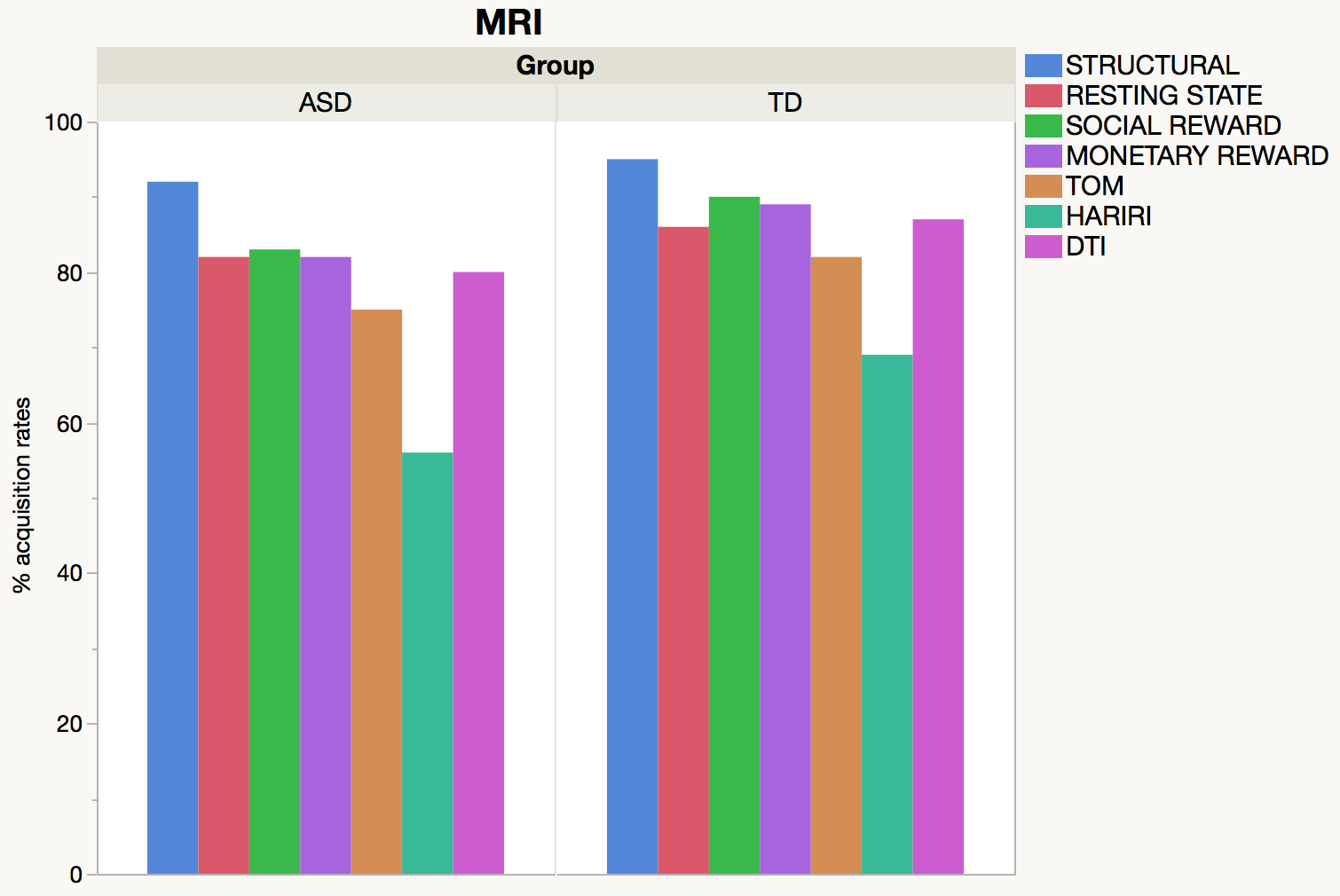


(b) by group and schedule


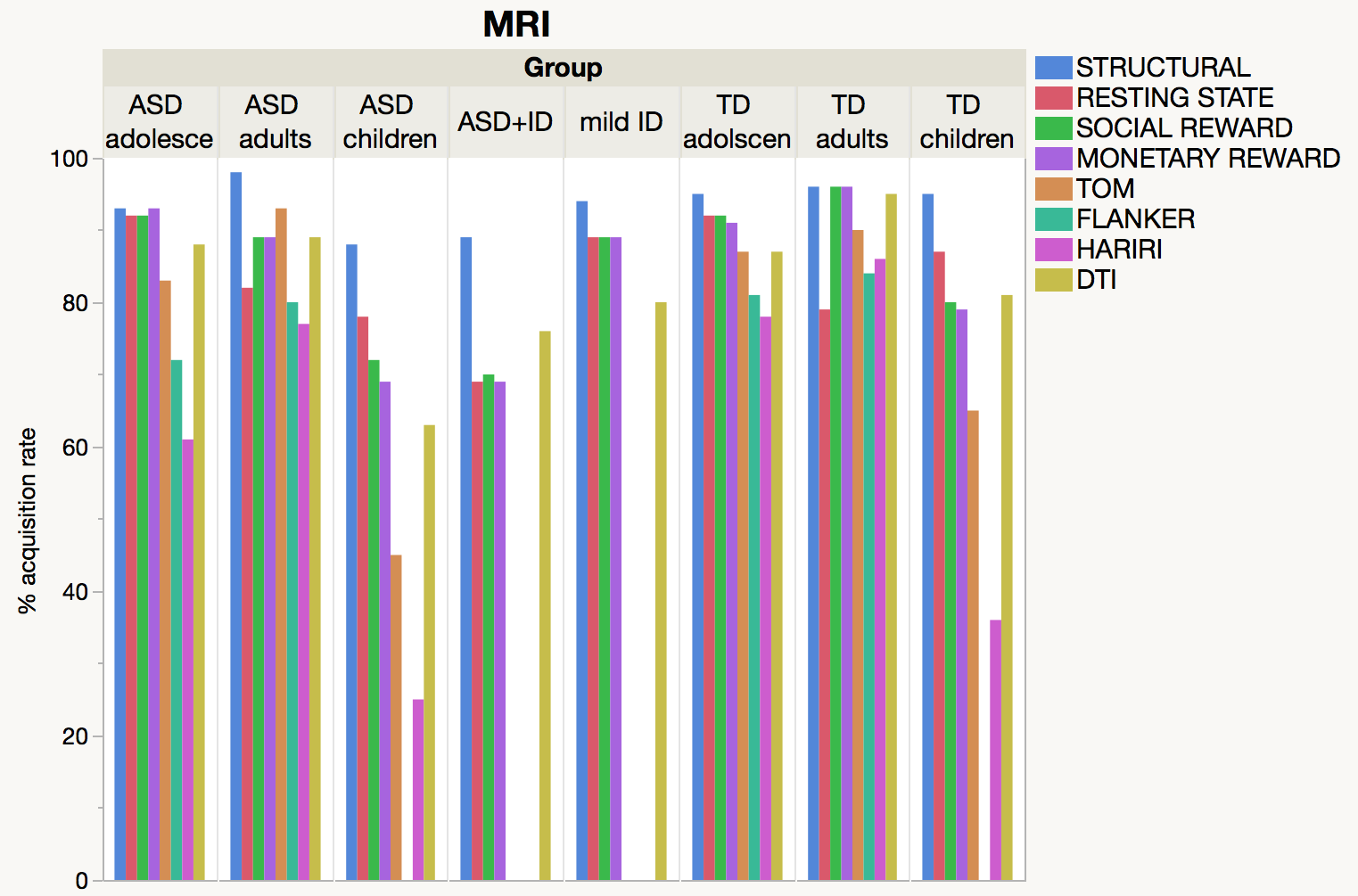


1. EEG , Note: EEG was not acquired at UCAM

(a) by group


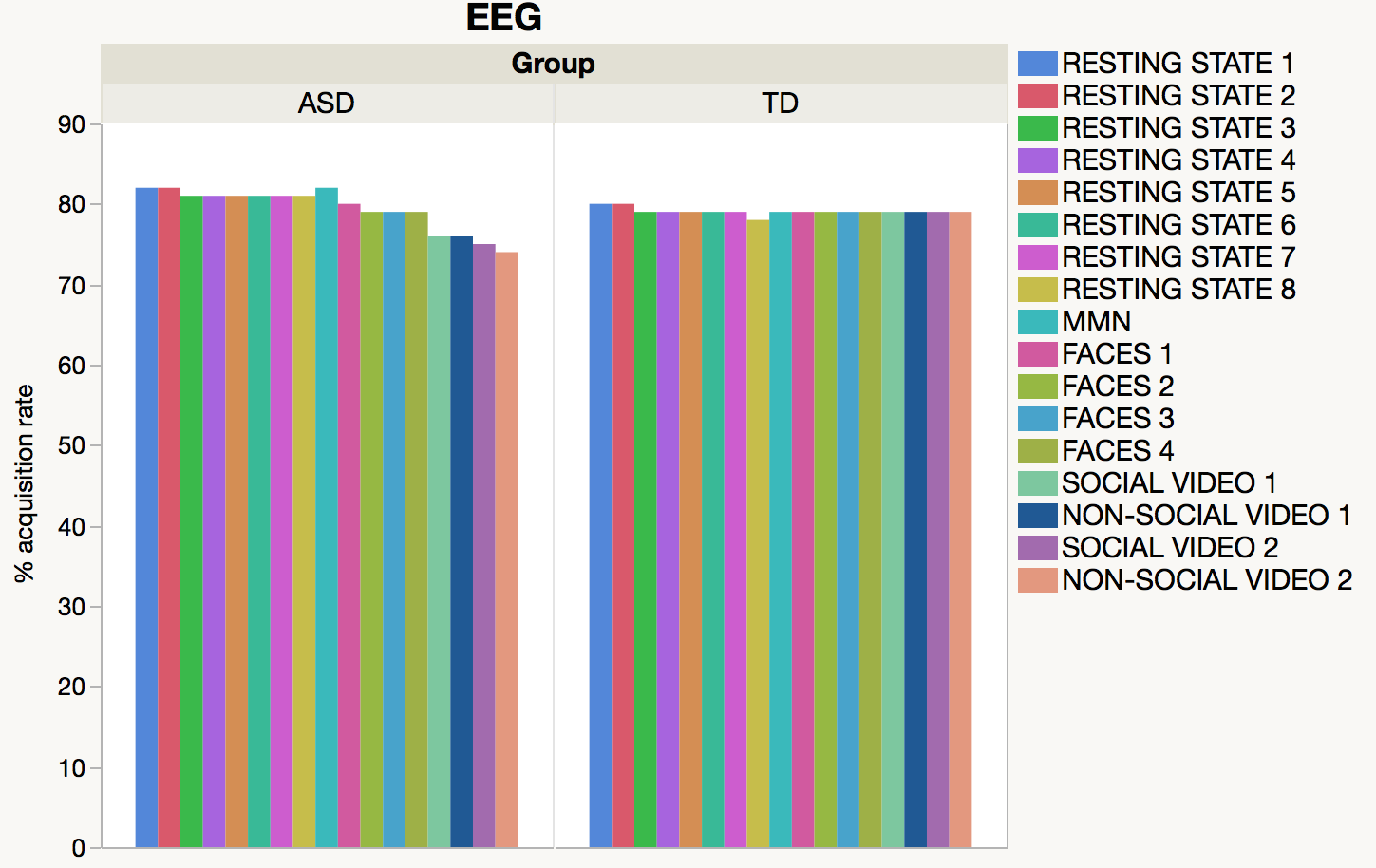


(b) by group and schedule


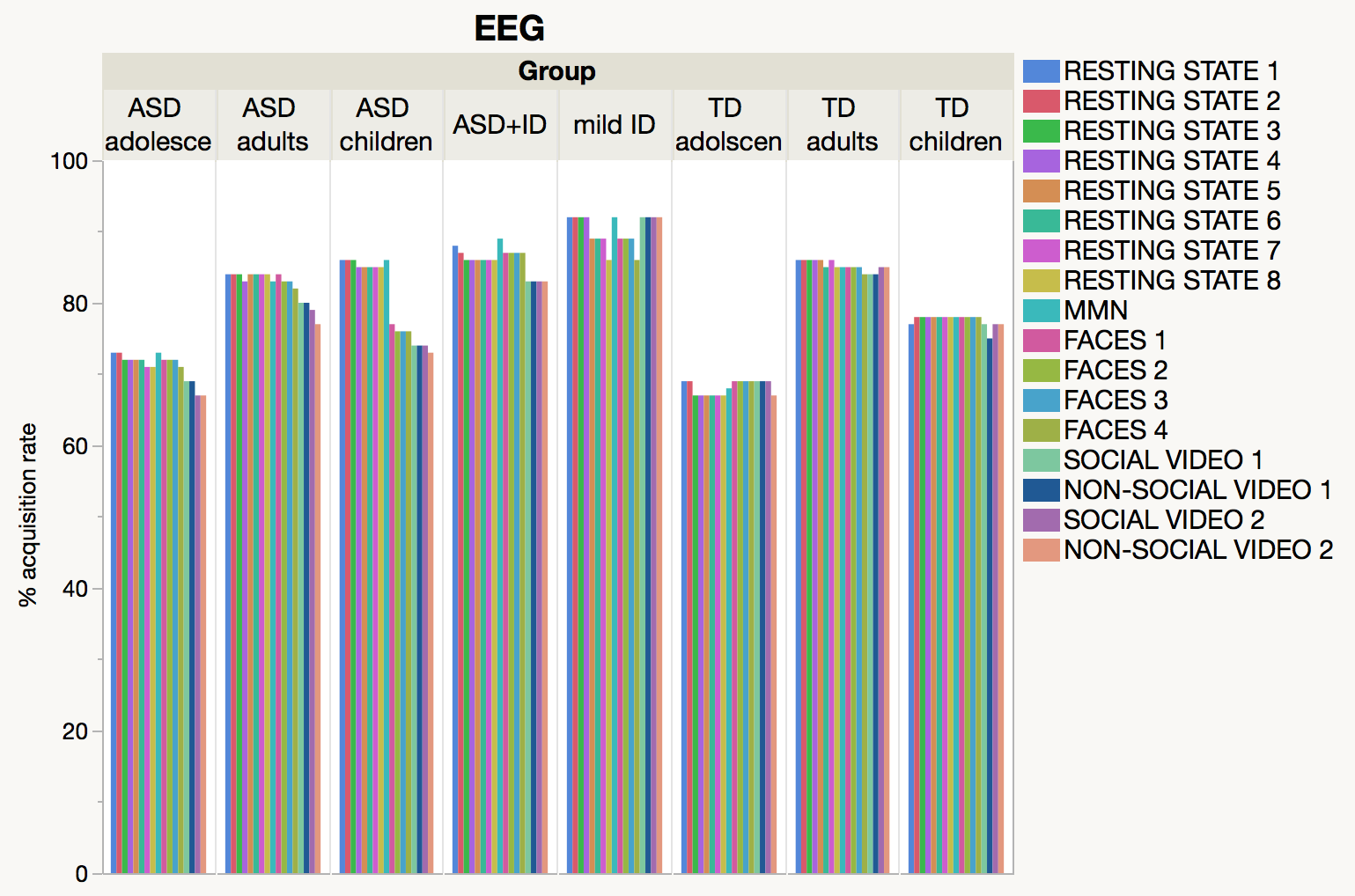


1. **Eye-tracking**

Note: Different stimuli (e.g., static social scenes, dynamic social scenes, biological motion, face pop-out, gap overlap) were presented in sets

(a) by group


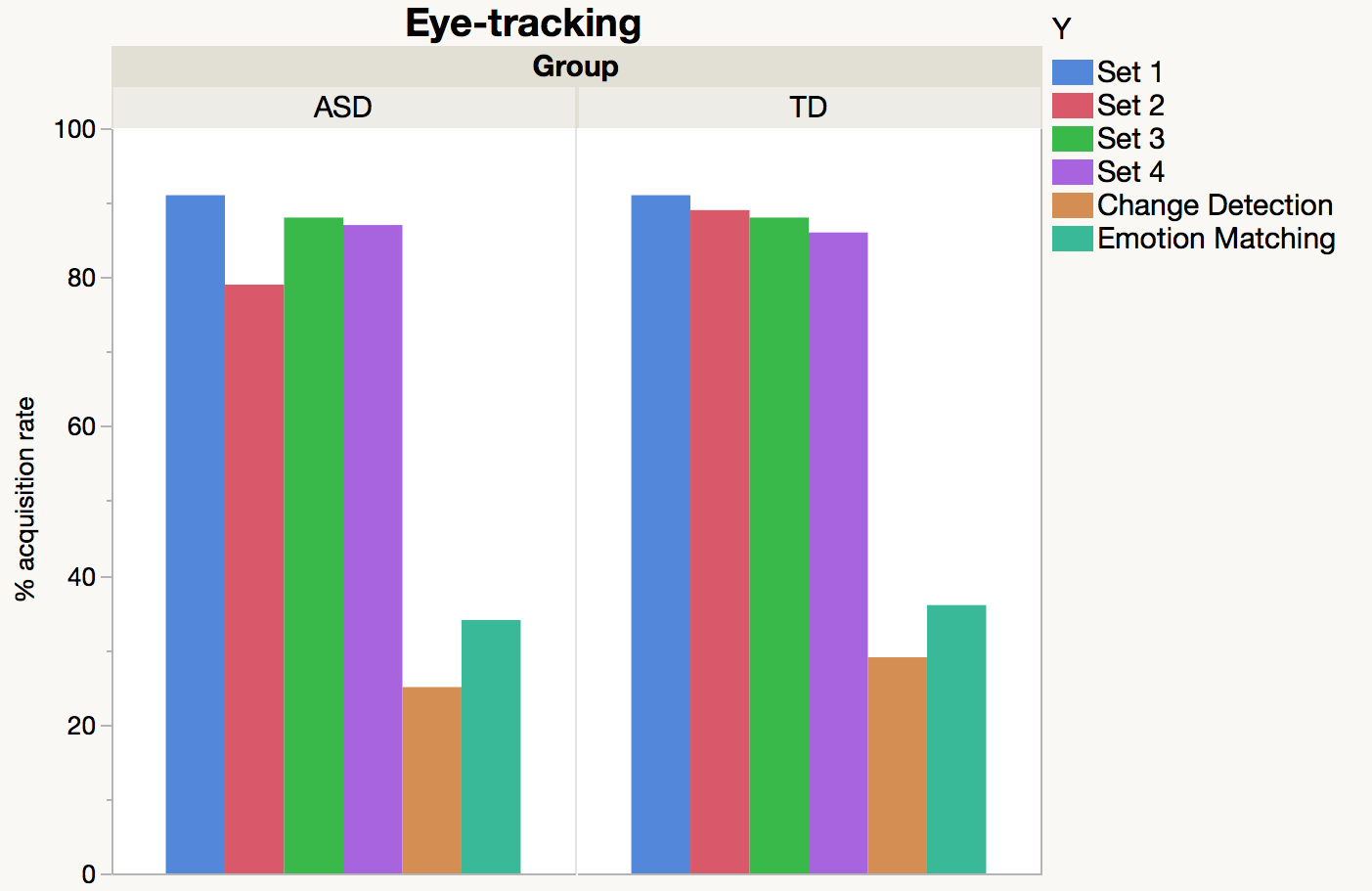


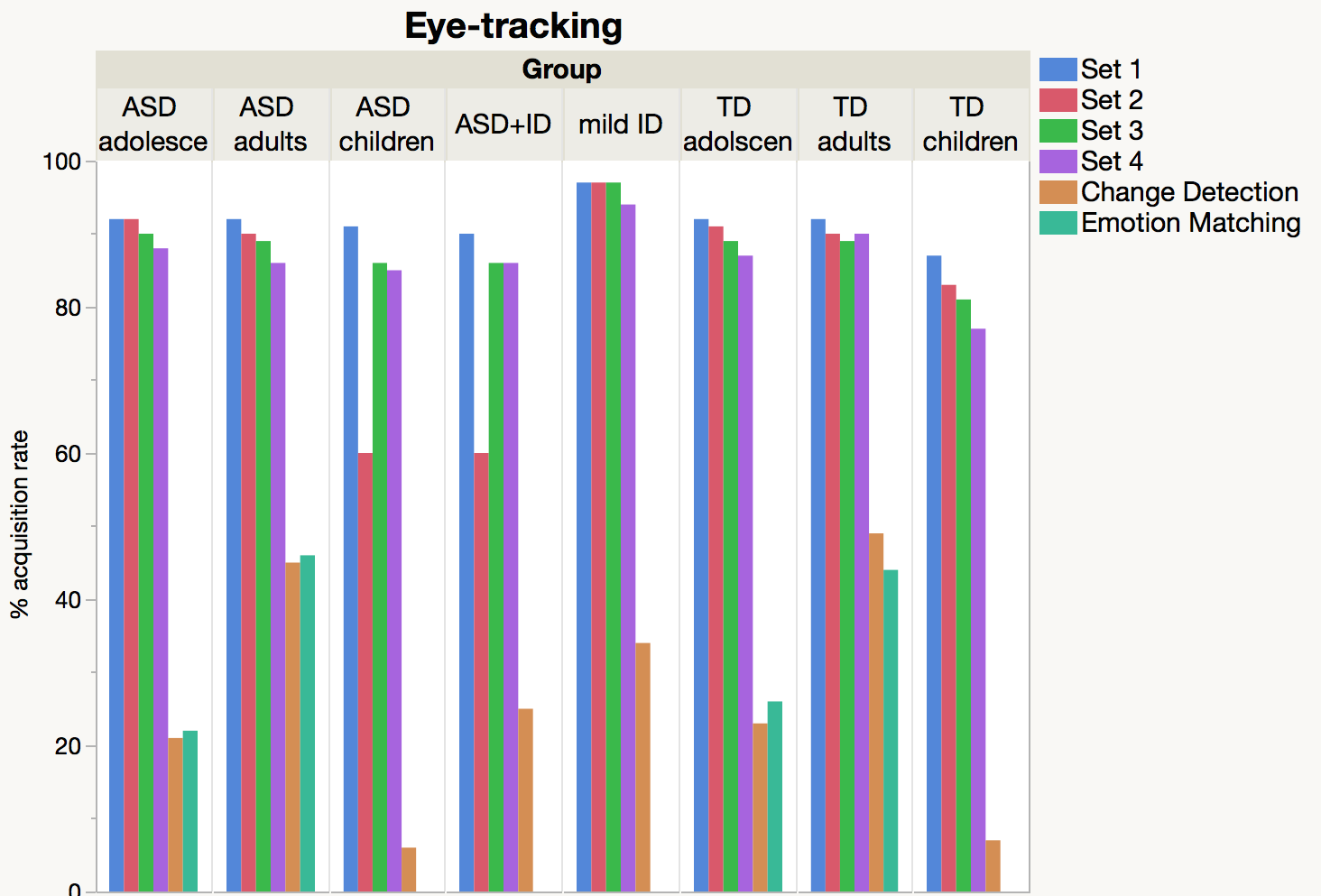


**4. Cognition**

(a) by group


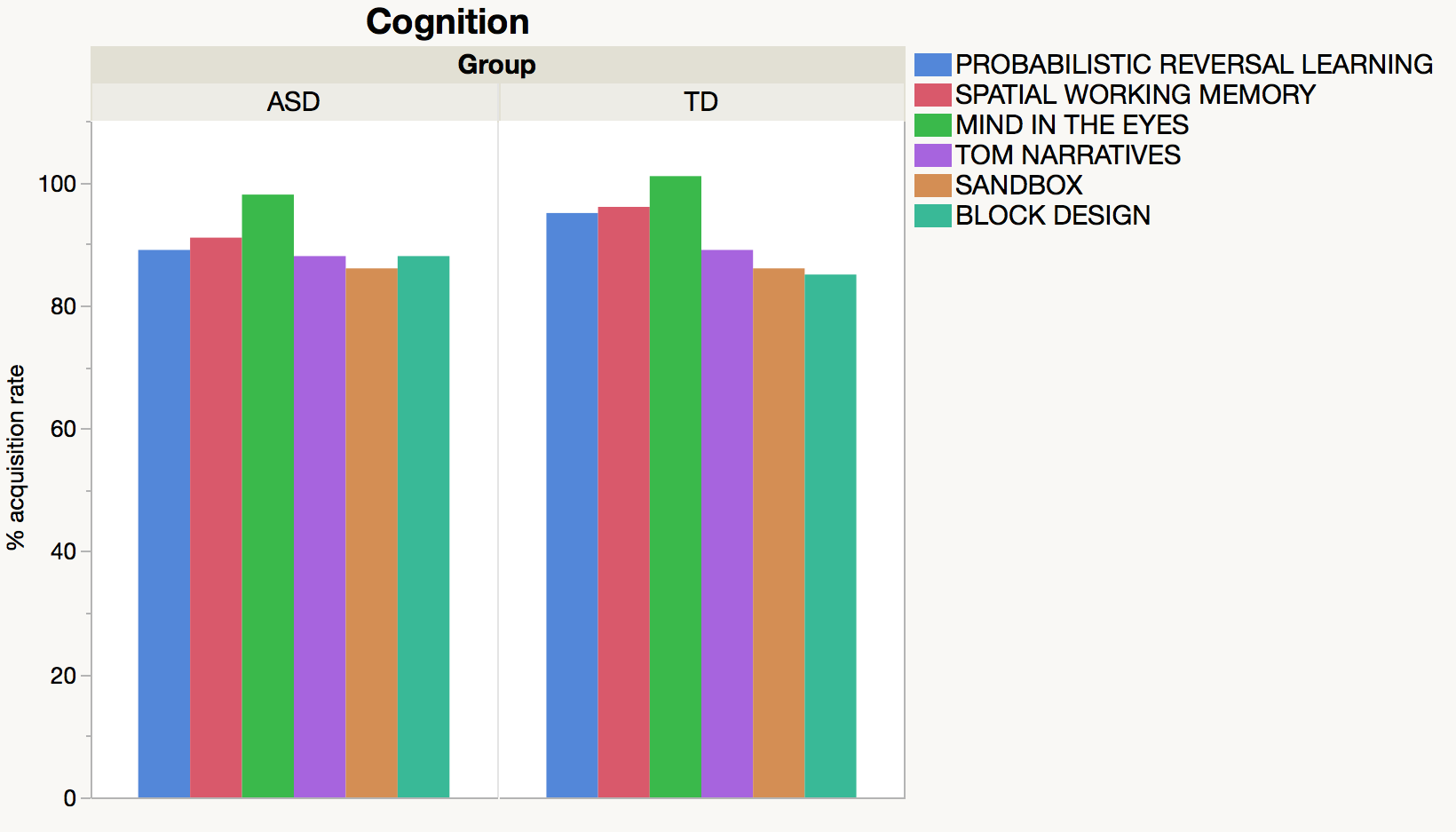


(b) by group and schedule


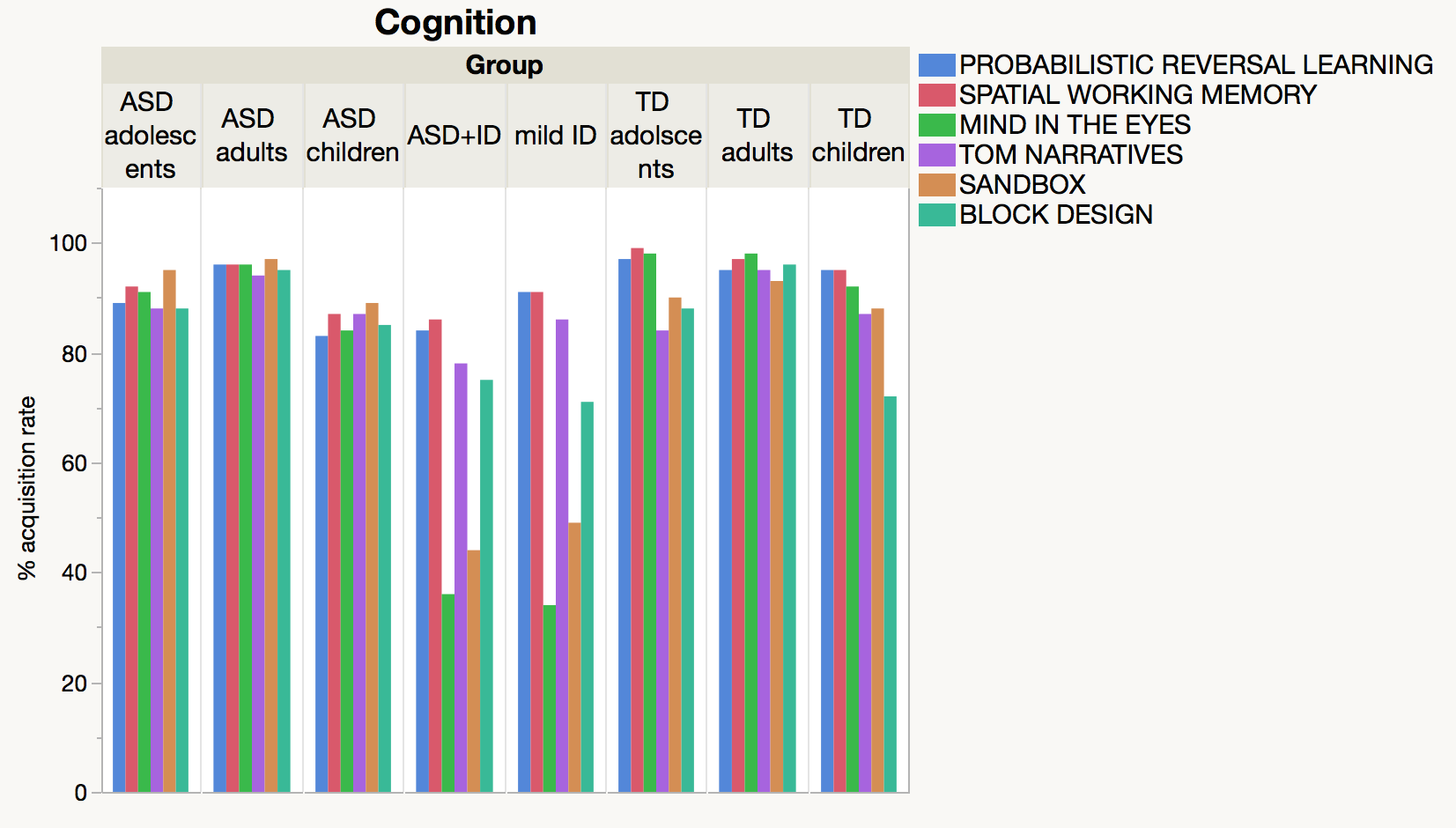


**6. Biosamples**

Blood and saliva sample collection are ongoing until the end of the FU assessment period (i.e., participants give one blood sample, either at baseline or at FU). Urine samples were only taken at baseline.

1. by group


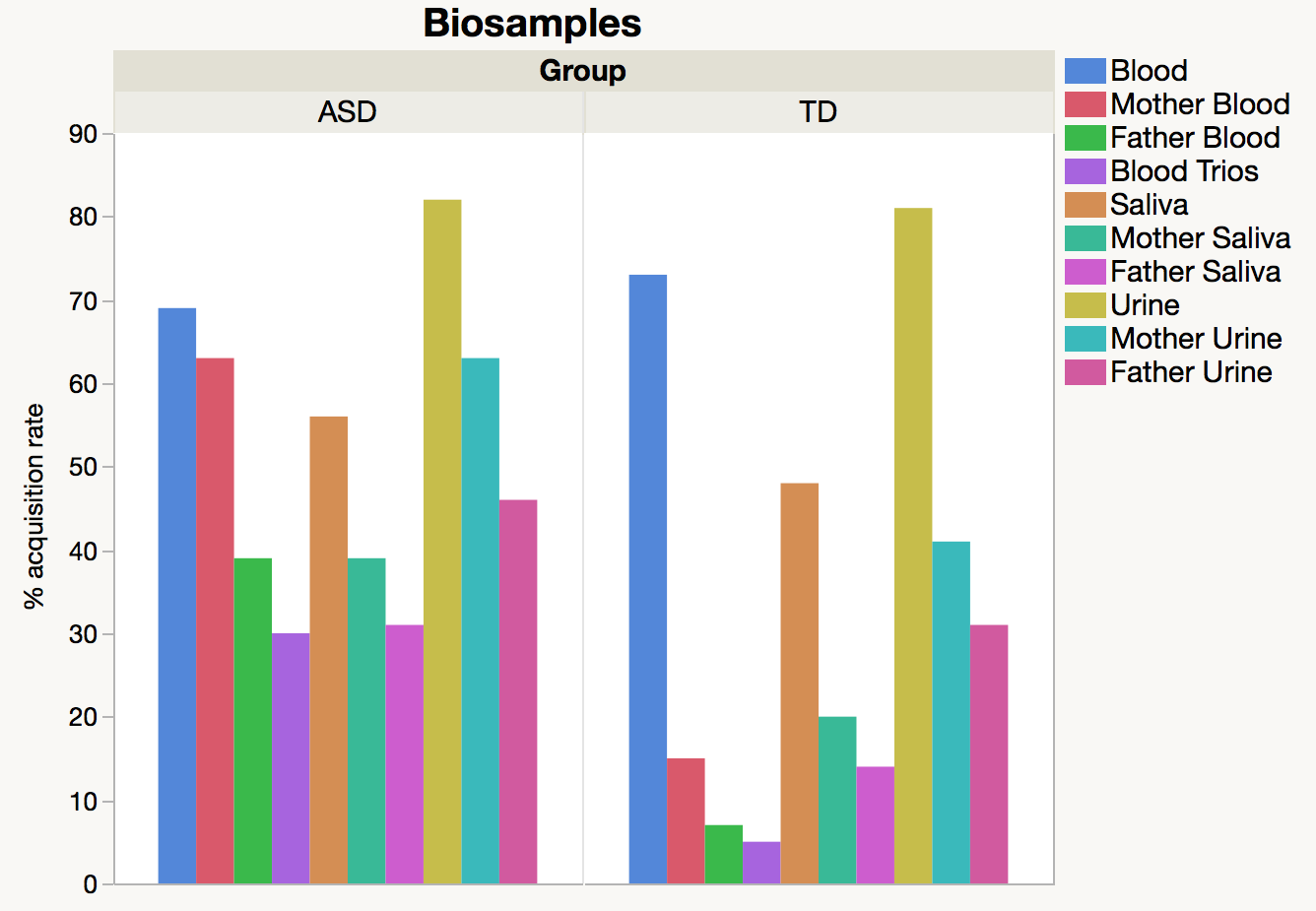


(b) by group and schedule
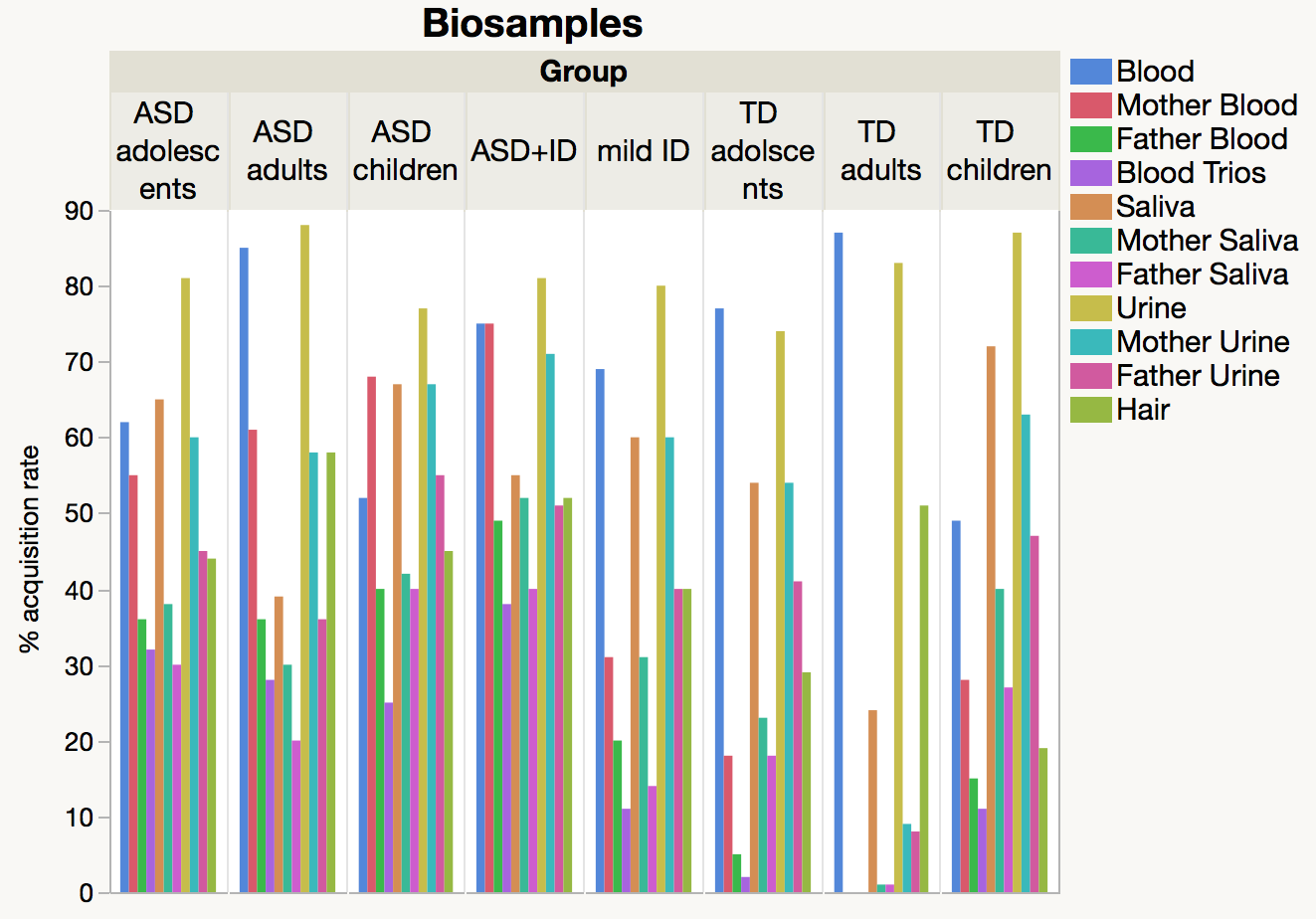

Supplement: Supplementary file 2 — Data acquisiton rates of baseline assessments, by data modality. (DOCX 786 kb) [file 13229_2017_146_MOESM2_ESM.docx]
