# Supplementary material for: The EU-AIMS Longitudinal European Autism Project (LEAP): design and methodologies to identify and validate stratification biomarkers for autism spectrum disorders
Source: Mol Autism. 2017 Jun 23;8:24. doi: 10.1186/s13229-017-0146-8 (PMC5481887; doi:10.1186/s13229-017-0146-8)
Supplement: Supplementary file 3 — Power calculations. (DOCX 1019 kb) [file 13229_2017_146_MOESM3_ESM.docx]

Additional file 3. Power calculations

1. Estimation of effect sizes(ES) at baseline and follow-up for the total cohort, accounting for 5-25% data loss, and for comparison of ASD-subgroups

|  | **ASD (N)** | **Control (N)** | **ES** |
| --- | --- | --- | --- |
| **Baseline** |  |  |  |
| Total | 390 | 255 | 0.23 |
| Total (-5% data loss) | 371 | 242 | 0.23 |
| Total (-25% data loss) | 293 | 191 | 0.26 |
| **Follow-up (12-24 months)** |  |  |  |
| Total | 312 | 204 | 0.25 |
| Total (-5% data loss) | 296 | 194 | 0.26 |
| Total (-25% data loss) | 234 | 153 | 0.29 |
| any subgroup N ~1/2 | 195 | 128 | 0.32 |
| any subgroup N ~1/3 | 130 | 85 | 0.39 |
| any subgroup N ~1/4 | 98 | 64 | 0.45 |

1. Power plots

| 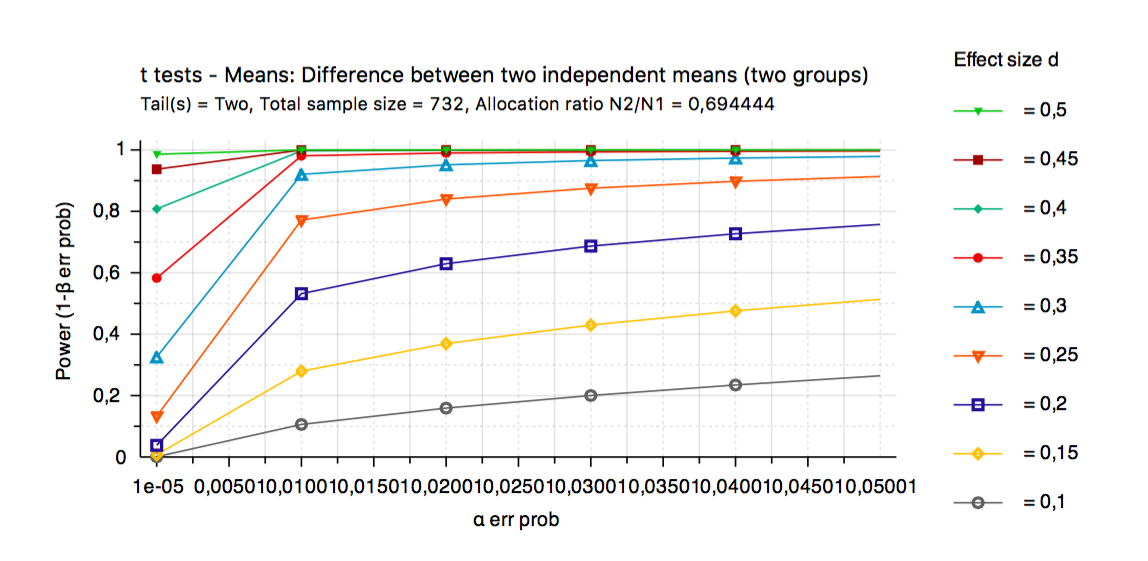 |  | 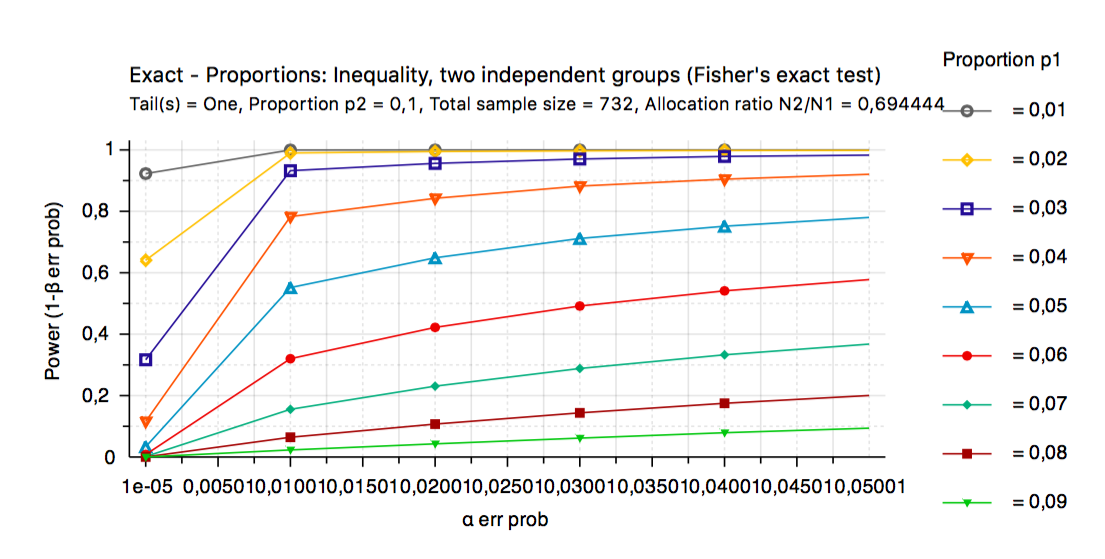 |  |
| --- | --- | --- | --- |
|  |  |  |  |
| 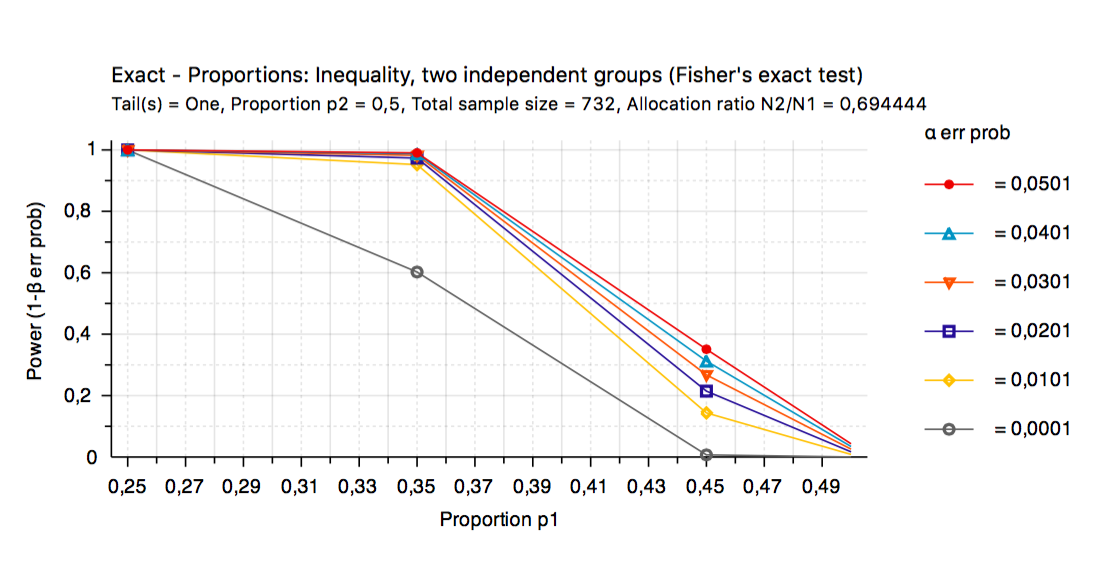 |  | 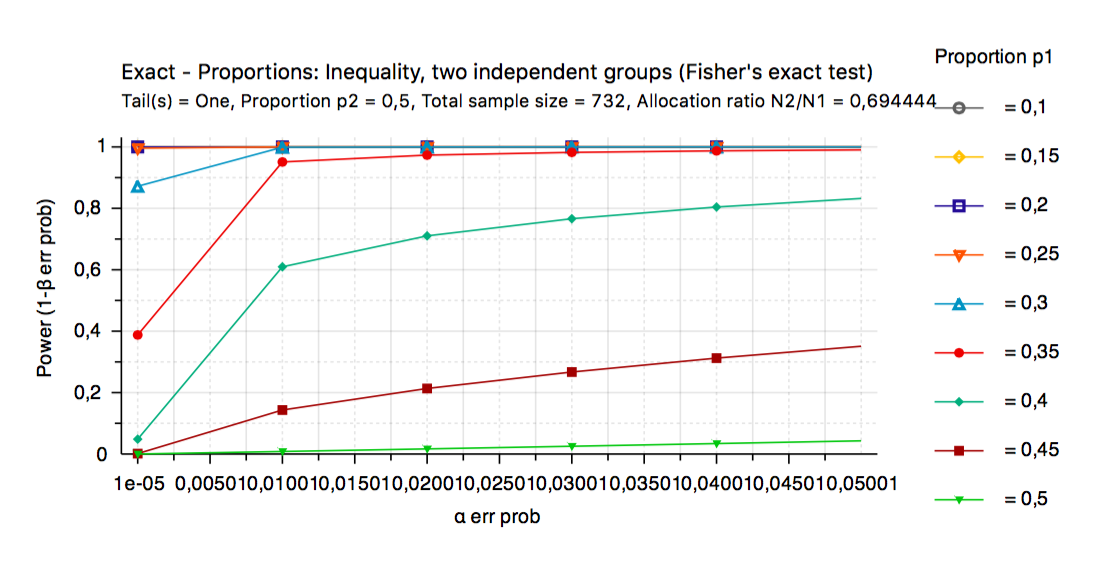 |  |
|  |  |  |  |
| 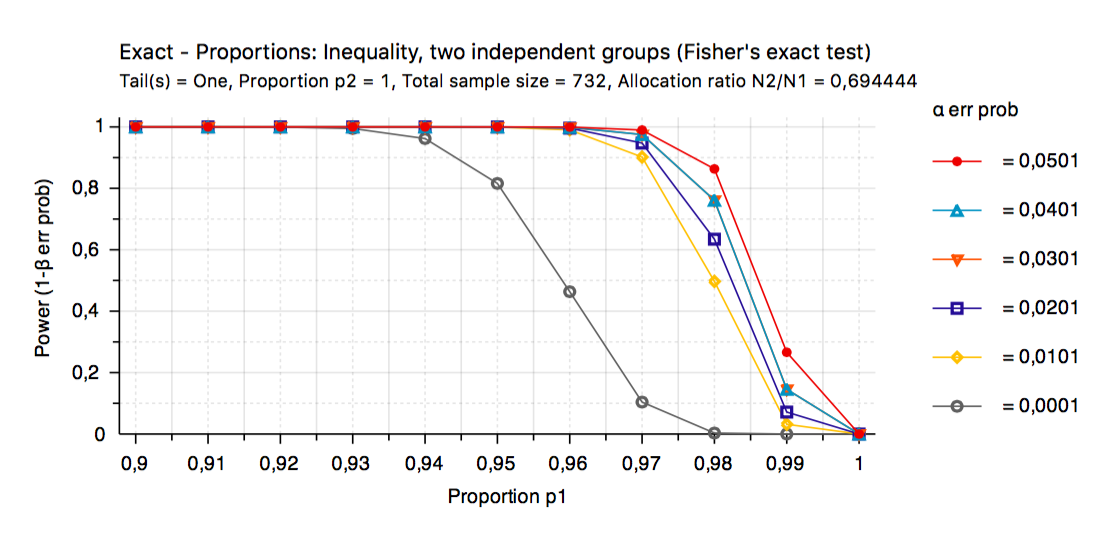 |  | 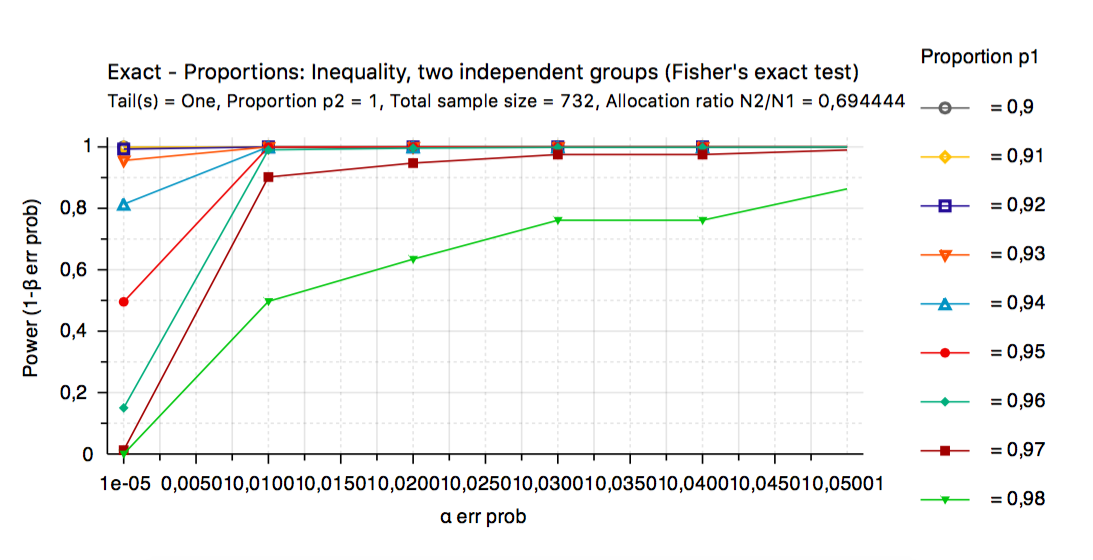 |  |
|  |  |  |  |
